# NGL-1/LRRC4C-Mutant Mice Display Hyperactivity and Anxiolytic-Like Behavior Associated With Widespread Suppression of Neuronal Activity

**DOI:** 10.3389/fnmol.2019.00250

**Published:** 2019-10-11

**Authors:** Yeonsoo Choi, Haram Park, Suwon Kang, Hwajin Jung, Hanseul Kweon, Seoyeong Kim, Ilsong Choi, Soo Yeon Lee, Ye-Eun Choi, Seung-Hee Lee, Eunjoon Kim

**Affiliations:** ^1^Center for Synaptic Brain Dysfunctions, Institute for Basic Science, Daejeon, South Korea; ^2^Department of Biological Sciences, Korea Advanced Institute for Science and Technology, Daejeon, South Korea

**Keywords:** NGL-1, LRRC4C, synaptic adhesion molecule, hyperactivity, anxiety, learning and memory, synaptic transmission, intrinsic neuronal excitability

## Abstract

Netrin-G ligand-1 (NGL-1), encoded by *Lrrc4c*, is a post-synaptic adhesion molecule implicated in various brain disorders, including bipolar disorder, autism spectrum disorder, and developmental delay. Although previous studies have explored the roles of NGL-1 in the regulation of synapse development and function, the importance of NGL-1 for specific behaviors and the nature of related neural circuits in mice remain unclear. Here, we report that mice lacking NGL-1 (*Lrrc4c^–/–^*) show strong hyperactivity and anxiolytic-like behavior. They also display impaired spatial and working memory, but normal object-recognition memory and social interaction. c-Fos staining under baseline and anxiety-inducing conditions revealed suppressed baseline neuronal activity as well as limited neuronal activation in widespread brain regions, including the anterior cingulate cortex (ACC), motor cortex, endopiriform nucleus, bed nuclei of the stria terminalis, and dentate gyrus. Neurons in the ACC, motor cortex, and dentate gyrus exhibit distinct alterations in excitatory synaptic transmission and intrinsic neuronal excitability. These results suggest that NGL-1 is important for normal locomotor activity, anxiety-like behavior, and learning and memory, as well as synapse properties and excitability of neurons in widespread brain regions under baseline and anxiety-inducing conditions.

## Introduction

Synaptic adhesion molecules play important roles in the regulation of synapse development, neural circuits, and behaviors (Shen and Scheiffele, 2010; Siddiqui and Craig, 2011; Um and Ko, 2013; de Wit and Ghosh, 2016; Südhof, 2017, 2018; Yuzaki, 2018; Kurshan and Shen, 2019). In line with these roles, synaptic adhesion molecules have been implicated in various brain dysfunctions, including autism spectrum disorders (ASDs), intellectual disability, schizophrenia, epilepsy, and addiction (Sudhof, 2008; Park and Biederer, 2013; Williams and Umemori, 2014; Ko et al., 2015; Kasem et al., 2018; Montani et al., 2019).

Netrin-G ligands (NGLs) belong to a family of synaptic adhesion molecules with three known members: NGL-1/LRRC4C, NGL-2/LRRC4, and NGL-3/LRRC4B (Lin et al., 2003; Kim et al., 2006; Woo et al., 2009b). NGL-1, NGL-2, and NGL-3 interact intracellularly with PSD-95, an abundant post-synaptic scaffolding protein (Sheng and Sala, 2001; Sheng and Hoogenraad, 2007; Sheng and Kim, 2011), and extracellularly with the presynaptic adhesion molecules, netrin-G1, netrin-G2, and LAR family receptor tyrosine phosphatases, respectively (Nakashiba et al., 2000, 2002; Yin et al., 2002; Lin et al., 2003; Kim et al., 2006; Woo et al., 2009a; Kwon et al., 2010; Seiradake et al., 2011). It has been suggested that the synaptic adhesions mediated by netrin-G1-NGL-1 and netrin-G2-NGL-2 complexes contribute to the development of distinct populations of neuronal synapses and neural circuits, based on the largely non-overlapping distribution of these two sets of proteins in distinct brain regions, synapses, and circuits (Nakashiba et al., 2002; Yin et al., 2002; Niimi et al., 2007; Nishimura-Akiyoshi et al., 2007; Woo et al., 2009b; Matsukawa et al., 2014; Choi et al., 2019). It is therefore expected that these two adhesion complexes may differentially influence distinct neural circuits and behaviors.

*In vivo* functions of NGLs in the regulation of neuronal synapses and behaviors have also been investigated, primarily using transgenic mice lacking NGL isoforms. NGL-1-mutant mice display suppressed excitatory synapse development and short-term plasticity in the hippocampus (Matsukawa et al., 2014; Choi et al., 2019). NGL-2-mutant mice show input-specific decreases in the development and function of hippocampal synapses (Denardo et al., 2012; Matsukawa et al., 2014; Um et al., 2018) and autistic-like social deficits and repetitive behaviors that are responsive to *N*-methyl-D-aspartate (NMDA) receptor activation (Um et al., 2018). NGL-3-mutant mice show impairments in brain development and hippocampal long-term depression involving altered Akt/GSK3β signaling, and altered locomotive and cognitive behaviors (Lee et al., 2019). However, whether NGL-1 deletion in mice impacts specific behaviors and related neural circuits remains unclear.

This is particularly important because NGL-1/LRRC4C has been implicated in various brain disorders, including bipolar disorder, ASD, and developmental delay. Specifically, it has been shown that *LRRC4C* (encoding NGL-1) is weakly associated with three types of temperaments—hyperthymic, dysthymic and cyclothymic—that enhance the risk of bipolar disorder (Greenwood et al., 2012). Moreover, an intergenic locus contiguous with *LRRC4C* is associated with the risk for ASD (Walker and Scherer, 2013), and an exonic deletion of *LRRC4C* is thought to act as a genetic modifier that promotes neurodevelopmental disorders, including a sensory processing disorder, apraxia, and autism (Maussion et al., 2017). In addition to NGL-1, the proteins that directly bind to NGL-1 have also been implicated in neuropsychiatric disorders. For instance, netrin-G1 is associated with Rett syndrome, ASD, schizophrenia and bipolar disorder (Borg et al., 2005; Archer et al., 2006; Nectoux et al., 2007; Eastwood and Harrison, 2008; Ohtsuki et al., 2008; O’Roak et al., 2012). Moreover, CDKL5, which phosphorylates NGL-1 to promote interactions between NGL-1 and PSD-95 and positively regulates excitatory synaptic structure and function (Ricciardi et al., 2012), is associated with Rett syndrome, epilepsy, intellectual disability, and ASD (Posar et al., 2015; Zhou et al., 2017; Zhu and Xiong, 2019).

To better understand *in vivo* functions of NGL-1 in the regulation of specific behaviors and neural circuits, we first characterized the behaviors of NGL-1-mutant mice. We found that these mice show strong hyperactivity, anxiolytic-like behavior, and learning and memory impairments. Immunostaining for c-fos, a marker of active neurons, in NGL-1-mutant mice under baseline and anxiety-inducing conditions revealed suppressed baseline and stress-induced neuronal activation in widespread brain regions. Some of these regions [anterior cingulate cortex (ACC), motor cortex, and hippocampal dentate gyrus] display distinctly altered synaptic transmission and intrinsic excitability. These results implicate NGL-1 in the regulation of specific behaviors, neural circuits, and synaptic and neuronal properties.

## Materials and Methods

### Animals

The *Lrrc4c^–/–^* mice used in this study have been previously described (Choi et al., 2019). Briefly, *Lrrc4c^–/–^* mice (LRRC4C^TM 1Lex^), obtained from The Mutant *Mouse* Resource and Research Center (MMRRC), were generated by introducing an NGL-1 targeting vector into 129/SvEvBrd-derived embryonic stem (ES) cells by homologous recombination, thereby replacing the third exon of the *Lrrc4c* gene encoding NGL-1 with a β-geo (LacZ/neo) cassette. These mice were mated with C57BL/6J albino mice, and the resulting F1 heterozygous mice were crossed with C57BL/6J mice for more than five generations to obtain *Lrrc4c^–/–^* mice in a C57BL/6J background. Mice were weaned at postnatal day 21, and mixed-genotype littermates of the same sex were housed together until experiments. All animals were fed *ad libitum* and housed under a 12-h light/dark cycle (light phase from 1:00 to 13:00). Mouse maintenance and procedures were performed in accord with the Requirements of Animal Research at KAIST, and all procedures were approved by the Committee of Animal Research at KAIST (KA2012-19), which oversees the ethics approval and consent required to perform rodent research at KAIST. For genotyping, the following primers were used. WT-for: GAACAAGATGACCTTACATCC, WT-rev: CAATAGGGTTGTTCCTCAACCAG, mut-for: CCCTAGGAATGCTCGTCAAGA, and mut-rev: CAGACTGTTTGAACTCCAGAAG (WT band size: 476 base pairs, mut band size: 289 base pairs).

### Behavioral Tests

All behavioral assays were performed using age-matched (2–5 months old) mice during light-off periods (active phase). Only male mice were used to eliminate effects of the estrous cycle of female mice. Before behavioral tests, mice were handled for at least 3 days (10 min/d), and at least 1-day-long rest periods were given between tests. Behavioral data were analyzed using Ethovision XT 10.1 software (Noldus), or manually, in a double-blind manner.

### Long-Term Mouse Movement Analysis

The Laboratory Animal Behavior Observation Registration and Analysis System (Laboras, Metris) was used to continuously monitor mouse movements in Laboras cages for 72 h. Grouped mice were transferred from their home cages and individually caged in newly bedded Laboras cages positioned on top of a vibration-sensitive platform. Only data from the last 48 h of recording was used in data analysis; the first day, which likely functions as a habituation period in a Laboras cage, was not included. The parameters analyzed were distance moved, climbing, and self-grooming. Data acquisition and analysis were performed using LABORAS 2.6 software (Metris).

### Open-Field Test

Mice were placed in a white acryl open field box (40 cm × 40 cm × 40 cm), and their movements were recorded for 60 min. Illumination was set at either 0 or 50 lux. Movements in the center arena (20 cm × 20 cm) and total distance moved and immobile time were measured using EthoVision XT 10.1 software (Noldus).

### Elevated Plus-Maze Test

The elevated plus-maze (EPM) consists of two open and two closed (30-cm walled) arms (5 cm × 30 cm each). Mice were placed in the center region of the maze facing the open arm. The open arm was illuminated at ∼200 lux, and the maze was situated 50 cm above the floor. Mouse movement was recorded for 10 min. Time spent in each set of arms and entering frequency into each arm were measured using Ethovision XT 10.1 software (Noldus).

### Light–Dark Test

The light–dark apparatus consists of open-roof, white (light) chamber connected to a closed, black (dark) chamber with an entrance that allows mice to freely move between the two chambers. The light chamber was illuminated at ∼250 lux. Mice were placed in the light chamber facing away from the entrance to the dark chamber, and their movements were recorded for 10 min. Entry into the light chamber was counted only when the mouse’s entire body crossed the entrance. The time spent in each chamber and number of entries into the light box were measured manually in a blinded manner.

### Marble-Burying Test

Marble-burying tests were performed as previously described (Deacon, 2006). Briefly, fresh bedding was added to a new cage to a depth of 5 cm, and 18 metal marbles (2-cm diameter) were placed with equal spacing (3 × 6 grid) on top of the bedding. Mice were placed individually in the described setting for 30 min at ∼50 lux. The number of buried marbles was manually counted afterward. A buried marble was defined as one that was covered by bedding over more than 70% of its initially exposed surface area.

### Forced Swim Test

For the forced swim test, a 2000 mL glass beaker was filled to 70% capacity with water (24–26°C) in a room illuminated at ∼85 lux. Subject mice were placed gently into the water, and their movements were recorded for 5 min. Immobility was defined as floating without any limb movements.

### Tail Suspension Test

For the tail suspension test, the tail of a subject mouse was inserted through a 1-mL pipette with its tip cut off at each end and then attached to a 20-cm stainless steel wire. The tail was then taped to the wire and the mouse was suspended by the apparatus at least 30 cm above the floor. Immobility was defined as hanging without any body movements. Immobility measurements started the moment the subject was suspended and ended after 5 min.

### Three-Chamber Test

The three-chamber test was performed essentially as described previously (Crawley, 2004; Nadler et al., 2004; Silverman et al., 2010). Mice were isolated for 3 days prior to the experiment. The experiment was performed in three sessions: habituation, S1 (social stranger 1) versus E (empty), and S1 (old social stranger) versus S2 (new social stranger). During the habituation session, mice were allowed to explore the center region for 5 min. Thereafter, S1 (129/SvJae strain), which underwent habituation to the chamber for 30 min per day before the test, was placed in a small container located in the corner of one side chamber; another small container in the opposite side chamber was left empty. Subject interactions were recorded for 10 min. Following the S1 versus E session, S2 (129/SvJae) was placed in the empty chamber, and subject interactions were recorded for 10 min. Sniffing and chamber time were analyzed using Ethovision XT 10.1 software (Noldus).

### Self-Grooming

Mice were individually placed into a new home cage without bedding for 20 min under dim-light (∼15 lux) conditions. The cage was covered with a transparent acrylic plate. The time spent grooming during the last 10 min was measured manually.

### Rotarod Test

Subjects were placed on a rotarod apparatus (Ugo Basile), and rotation speed was increased from a starting speed of 4 to 40 rpm over 5 min. The assay was performed on 5 consecutive days, and the latency to falling from the rod was measured.

### Pre-pulse Inhibition

Acoustic startle responses were measured using the Med Associates Startle Reflex System (St. Albans). The device consists of a metal cage positioned atop a series of piezoelectric platforms and a sound system for inducing acoustic startle, both of which are located inside a sound-attenuating chamber. Each platform was calibrated using a spinner-type calibrator (Med Associates Startle Calibrator). Mice were placed within the metal cage and habituated to the system for 5 min in the presence of white background noise. Then, 48 trials were performed in series, with each trial presenting a null, startle, prepulse + startle, or prepulse-only stimulus. Trial-by-trial intervals and the order of trials according to stimulus were set beforehand in a pseudorandom manner. Prepulse stimuli were set at sound intensities of 74, 82, or 90 dB; the startle stimulus was set at 120 dB.

### Delayed Non-matching T-Maze

This test consists of three phases: food deprivation, habituation to the T-maze, and reward alteration. During the food-deprivation period, mice feeding was restricted to only small chow pieces (1.5 g/mouse), placed in one end of the cage daily, until mouse weights decreased to between 80 and 85% of starting weights. Thereafter, mice were introduced into the T-maze for habituation. All mice from the same home cage were placed together in the T-maze for 3 min with both arm doors opened and a reward (1:1 diluted sweetened milk) at each arm end. This was repeated twice per day, with at least 10-min intervals between exposures, for 2 days. One the third day of habituation, each individual was subjected to runs from the starting arm with one arm open and the other arm blocked by its door. Equal numbers of left and right runs were given in a random, non-alternating order. After habituation to the T-maze, the task was started. The task was divided into forced and choice runs. In the forced run, one arm was closed so that the subject was compelled to end up in the other arm. After the subject finished 50 μl of reward, it was returned to the start box. After a 20-s delay, the mouse was released from the start box and allowed to freely choose either arm. If correct, the mouse was allowed time to finish its reward and then was gently placed back in its cage. If incorrect, the mouse was constrained in the wrong arm for 15 s as a punishment. The time interval between trials was greater than 20 min. The experiment proceeded until wild-type (WT) mice achieved a success rate of 90%.

### Morris Water Maze

The maze is a circular tank 100 cm in diameter, containing water maintained at 22–25°C. White ink was dissolved in the water until the liquid became opaque enough to conceal the platform present under the water. Three trials were performed per subject per day. In each trial, mice were placed in three different quadrants in a random order. If the subject failed to reach the hidden platform within the 1-min testing period, it was guided to the platform and removed from the maze after a compulsory 15-s delay on the platform. For the probe test, mice were placed at the center of the maze, and their movements were recorded for 1 min. Time spent in each quadrant and number of crossings over the previous position of platform were analyzed using Ethovision XT 10.1 software (Noldus). For the reversal test, the platform was placed in the opposite quadrant, and the procedure was repeated until WT mice reach the platform within 20 s. After reversal learning phases, another probe test for reversal learning was performed in the same manner.

### Novel-Object–Recognition Test

One day prior to the novel-object–recognition test, the subject mouse was habituated to a 40 cm × 40 cm × 40 cm open-field box for 1 h. The test was divided into 2 days. On day 1, the subject was placed in the chamber with two identical objects 20 cm apart from each other in the middle of the chamber for 10 min. On day 2, one of the two objects was replaced with a novel object (side preference was avoided by randomly choosing the side of objects in each trial), and exploration of the two objects by the mouse was measured manually. Preference for the novel object was defined as the time spent sniffing the novel object divided by the total amount of time spent sniffing both objects.

### Visual Discrimination Test

Mice were trained under the visual discrimination task in the head-fixed condition (Lee et al., 2012). Mice were first implanted with head-plates on the skull and recovered for at least a week before starting the task. Mice then were mildly deprived of water and trained in the head-fixed condition to lick to get the water reward. The weight of mice was measured daily not to be less than 80% with at least 1 ml of water per day. The training started with free water with the lick and moved to the conditioning of the Go visual stimuli (vertical orientation drifting gratings) with the water reward. After 3–5 days of conditioning, mice were trained under the discrimination task, in which the No-go visual stimuli (horizontal orientation) were randomly presented with the Go stimuli. If mice licked after the No-go stimulus, a mild air-puff and the time-out delay were given as a punishment. Mice were trained daily with 400–800 trials (until mice show more than three times of miss in a row) per day, and the discriminability (d′) was measured daily by quantifying the divergence of lick rates under the Go and the No-Go stimuli. After mice reach the d′ > 2.5, lower contrasts (20, 40, and 60%) of visual stimuli were given in a random order at equal ratio. For the visual stimulation, 8-inch LCD monitor (120 lux maximum luminance, gamma corrected with custom software) was located 15 cm from the left eye, and full-field vertical and horizontal drifting gratings (100% contrast, 2 Hz, 0.04 cycles per degree, up to 4 s) were presented.

### c-Fos Analysis

Mice in the experimental group (EPM exposed) were placed in the open arm of the EPM apparatus at ∼200 lux for 10 min. Thereafter, they were placed individually in a newly bedded cage in a dark room for a 90-min rest period to maximize c-fos expression. Mice in the control group were individually placed in a newly bedded cage in a dark room for 10 min (without EPM exposure) and underwent a 90-min habituation period in the same cage. After anesthetization and cardiac perfusion, coronal or horizontal sections of the brains were prepared, and the five most representative sets across the rostral-caudal axis of coronal sections (four adjacent sections per set) and one horizontal section for the ventral hippocampus were selected for c-fos staining (Santa Cruz sc-52, 1:1000). All images were acquired using a slide scanner (Axio Scan.Z1). Regions of interest (ROIs) were manually selected according to the Allen Brain Atlas, and the number of c-fos-positive cells was counted using Image J software. Images of c-fos staining were thresholded, and c-fos-positive cells greater than 10 pixels in size with a circularity greater than 0.01 were counted. Cell count values were normalized to ROI areas.

### Brain Slices for Electrophysiology

Acute horizontal brain slices for DG electrophysiology were obtained by anesthetizing 2–4-month-old adult male mice with isoflurane (Terrell, Piramal Critical Care) and extracting the brain into a 0°C dissection buffer consisting of, in mM: 212 sucrose, 25 NaHCO_3_, 5 KCl, 1.25 NaH_2_PO_4_, 10 D-glucose, 2 sodium pyruvate, 1.2 sodium ascorbate, 3.5 MgCl_2_, 0.5 CaCl_2_ and bubbled with 95% O_2_/5% CO_2_. The dorsal part of the brain was fixated with cyanoacrylate glue onto a triangular agar gel with a 12 degree angle and transferred to a vibratome (VT1200s, Leica), where horizontal brain sections were obtained (Remondes and Schuman, 2002). To obtain dorsal hippocampal sections, hemi-sectioned brains were glued along the midline surface. Brains were sliced at a thickness of 400 μm, and resulting slices were transferred to a 32°C holding chamber containing a solution of artificial cerebrospinal fluid (aCSF; in mM: 125 NaCl, 25 NaHCO_3_, 2.5 KCl, 1.25 NaH_2_PO_4_, 10 D-glucose, 1.3 MgCl_2_, 2.5 CaCl_2_). Slices were recovered at 32°C for 1 h, and afterward further recovered in room temperature (20–25°C) for 30 min. Once recovery was finished, slices were transferred to a recording chamber, where all electrophysiological experiments were performed at 28°C with circulating aCSF. Cells were visualized under differential interference contrast illumination in an upright microscope (B50WI, Olympus). Acute coronal brain slices for the ACA and MO regions were obtained following the protective recovery method (Ting et al., 2014). In brief, mice were anesthetized with an intraperitoneal injection of a ketamine-xylazine cocktail, followed by transcardiac perfusion of a protective buffer (NMDG aCSF) consisting of, in mM: 100 NMDG, 12 NAC, 30 NaHCO_3_, 20 HEPES, 25 glucose, 2 thiourea, 5 Na-ascorbate, 3 Na-pyruvate, 2.5 KCl, 1.25 NaH_2_PO_4_, 0.5 CaCl_2_, and 10 MgSO_4_. After clearing of the blood, the brain was extracted and the cerebellum dissected out. Then, the brain was fixated with cyanoacrylate glue onto the platform and transferred to the vibratome. Brains were sliced at a thickness of 400 μm, and the resulting brain slices transferred to a 32°C holding chamber containing NMDG aCSF for 13 min. After the incubation, the slices were transferred and recovered in a chamber containing a recovery buffer consisting of, in mM: 92 NaCl_2_. 12 NAC, 30 NaHCO_3_, 20 HEPES, 25 glucose, 2 thiourea, 5 Na-ascorbate, 3 Na-pyruvate, 2.5 KCl, 1.25 NaH_2_PO_4_, 2.5 CaCl_2,_ and 1.3 MgCl_2_. Once recovery was finished, slices were transferred to a recording chamber, where all electrophysiological experiments were performed at 28°C with circulating aCSF.

### Whole-Cell Recording

For whole-cell voltage-clamp recordings, thin-walled borosilicate capillaries (30-0065, Harvard Apparatus) were used to make pipettes with resistance 2.3∼3.5 MΩ via a two-step vertical puller (PC-10, Narishige). For mEPSC and sEPSC recordings, pipettes were filled with an internal solution composed of, in mM: 117 CsMeSO_4_, 10 EGTA, 8 NaCl, 10 TEACl, 10 HEPES, 4 Mg-ATP, 0.3 Na-GTP, 5 QX-314. For mIPSC and sIPSC recordings, the internal solution contained, in mM: 115 CsCl, 10 EGTA, 8 NaCl, 10 TEACl, 10 HEPES, 4 Mg-ATP, 0.3 Na-GTP, 5 QX-314. For the measurement of intrinsic neuronal properties, pipettes were filled with an internal solution containing, in mM, 137 potassium gluconate, 5 KCl, 10 HEPES, 0.2 EGTA, 10 sodium phosphocreatine, 4 Mg-ATP, 0.5 Na-GTP. All internal solutions were titrated to pH 7.35 and adjusted to an osmolarity of 285 mOsm. For mEPSC experiments, 60 μM picrotoxin and 0.5 μM tetrodotoxin (Tocris) were added. For mIPSC experiments 10 μM NBQX (Tocris), 50 μM D-AP5 (Tocris), 0.5 μM tetrodotoxin (Tocris) were added. For sEPSC experiments, 60 μM picrotoxin was added. For sIPSC experiments 10 μM NBQX (Tocris), and 50 μM D-AP5 (Tocris) were added. For neuronal property experiments, 10 μM NBQX (Tocris), 50 μM D-AP5 (Tocris), and 60 μM picrotoxin. Access resistance was maintained as to be no greater than 20 MΩ or else excluded from data acquisition. Signals were filtered at 2 kHz and digitized at 10 kHz under the control of Multiclamp 700B Amplifier (Molecular Devices) and Digidata 1550 Digitizer (Molecular Devices). Cells were approached with the internal solution-filled pipette to make a giga seal, after which cells were gently ruptured via suction and maintained thereafter at −70 mV. After voltage-clamped cells were stabilized (∼3 min post-rupture), recordings were obtained. Access resistance was monitored throughout the stabilization period and immediately before and after the data acquisition. The acquired data were analyzed using Clampfit 10 (Molecular Devices).

### Experimental Design and Statistical Analysis

All quantitative analyses were performed using age-matched WT (C57BL/6J) and *Lrrc4c^–/–^* mice. For electrophysiological analyses, three to five animals were used in each experiment. For behavioral assays, at least two independent cohorts were used, and behavioral assays were implemented in order of increasing stress, although not all behavioral results were derived from the same sets of cohorts. For c-fos imaging, up to four slices per mouse per section were used, to a maximum of 16 slices per section. Statistical outliers were removed using Grubb’s test. Normality and equal variance were tested for all statistical analyses using the D’Agostino-Pearson omnibus normality test. Sample size was determined by the nature of the experimental design. Statistical tests were performed using Prism 5 (GraphPad) and SigmaPlot 12.0 (Systat Software). Two-way analysis of variance (ANOVA) was performed for tests of both genotype and any additional factor; *post hoc* analyses were performed only when either the interaction or both main factors were significant.

## Results

### *Lrrc4c^–/–^* Mice Display Hyperactivity in a Familiar Environment

*Lrrc4c*^–/–^ (KO) mice displayed largely normal physiological traits as compared to WT counterparts. They were born in numbers as expected by the Mendelian ratio. No obvious physical changes were present other than a slight but not statistically significant decrease in average body weights (WT = 29.2 g, KO = 28.9 g at 4 months). There were no differences in early survival or fertility of heterozygous (*Lrrc4c*^±^) mice, which were used for production of *Lrrc4c^–/–^* mice.

**FIGURE 1:**
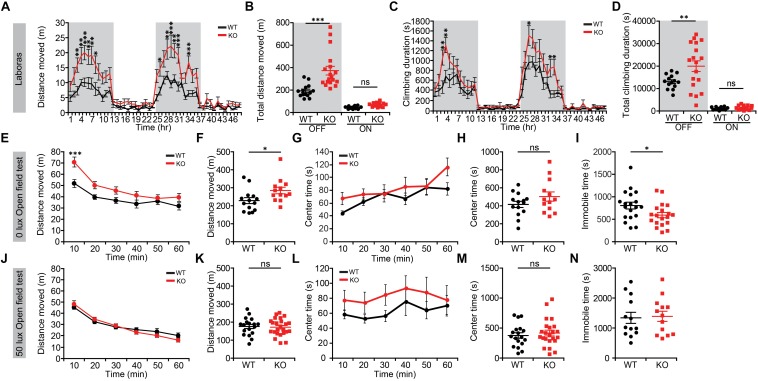
*Lrrc4c^–/–^* mice display hyperactivity in a familiar environment. **(A,B)** Increased locomotor activity of *Lrrc4c^–/–^* mice (2–5 months; male) in Laboras cages (familiar environment), as shown by distance moved during the last two consecutive days. *n* = 14 mice (WT), *n* = 18 (KO), ^∗^*p* < 0.05, ^∗∗^*p* < 0.01, ^∗∗∗^*p* < 0.001, two-way repeated-measures ANOVA with Holm-Sidak multiple comparison test [**(A)** interaction, *F*_(__47__,__1410__)_ = 3.206, *p* < 0.0001; time, *F*_(__47__,__1410__)_ = 24.99, *p* < 0.0001; genotype, *F*_(__1__,__30__)_ = 18.23, *p* = 0.0002; 3 h, *p* = 0.0072; 4 h, *p* = 0.0041; 5 h, *p* = 0.0007; 6 h, *p* = 0.0055; 7 h, *p* = 0.0016; 8 h, *p* = 0.0169; 26 h, *p* = 0.0483; 28 h, *p* = 0.0055; 29: *p* < 0.0001; 30 h, *p* = 0.0023; 31 h, *p* = 0.0003; 34 h, *p* = 0.0002; **(B)** interaction, *F*_(__1__,__60__)_ = 11.36, *p* = 0.0013; genotype, *F*_(__1__,__60__)_ = 19.99, *p* < 0.001; time, *F*_(__1__,__60__)_ = 96.26, *p* < 0.001; OFF, *p* = 0.4393, ON, *p* < 0.001]. **(C,D)** Increased climbing of *Lrrc4c^–/–^* mice (2–5 months; male) in Laboras cages, as shown by climbing duration. *n* = 14 mice (WT), *n* = 18 (KO), ^∗^*p* < 0.05, ^∗∗^*p* < 0.01, two-way repeated-measures ANOVA with Holm-Sidak multiple comparison test [**(C)** interaction, *F*_(__47__,__1410__)_ = 1.859, *p* = 0.0004; time, *F*_(__47__,__1410__)_ = 27.58, *p* < 0.0001; genotype, *F*_(__1__,__30__)_ = 6.135, *p* = 0.019; 3 h, *p* = 0.0056; 4 h, *p* = 0.0018; 27 h, *p* = 0.0163; 33 h, *p* = 0.0239; 34 h, *p* = 0.0157; **(D)** interaction, *F*_(__1__,__60__)_ = 5.357, *p* = 0.0241; genotype, *F*_(__1__,__60__)_ = 6.568, *p* = 0.0129; time, *F*_(__1__,__60__)_ = 116.3, *p* < 0.001; OFF, *p* = 0.8612, ON, *p* = 0.0021]. **(E–I)** Modestly increased locomotor activity of *Lrrc4c^–/–^* mice (2–5 months; male) in the open-field test at 0 lux, as shown by distance moved. Note that the center time, a measure of anxiety-like behavior, is not altered and that immobile time is decreased in *Lrrc4c^–/–^* mice, in line with the hyperactivity in *Lrrc4c^–/–^* mice. *n* = 14 (WT), n = 13 (KO), ^∗^*p* < 0.05, ^∗∗∗^*p* < 0.001, ns, not significant, two-way repeated-measures ANOVA with Holm-Sidak multiple comparison test, Mann–Whitney test **(F)** and Student’s *t*-test **(H)** [**(E)** interaction, *F*_(__5__,__125__)_ = 4.211, *p* = 0.0014; genotype, *F*_(__5__,__125__)_ = 5.288, *p* = 0.0301; time, *F*_(__1__,__25__)_ = 5.288, *p* < 0.001; 10 min, *p* = 0.0006; **(F)** *U* = 41; *p* = 0.0145; **(G)** *F*_(__5__,__200__)_ = 2.606, *p* = 0.0262; genotype, *F*_(__1__,__40__)_ = 0.0756, *p* = 0.7848; time, *F*_(__5__,__200__)_ = 127.5, *p* < 0.001; **(H)** *t*_(__40__)_ = 0.2749; *p* = 0.7848; **(I)** *t*_(__36__)_ = 2.216; *p* = 0.0331]. **(J–N)** Normal locomotor activity of *Lrrc4c^–/–^* mice (2–5 months; male) in the open-field test at 50 lux, as shown by distance moved. Note that the center time and immobile time are not altered. *n* = 18 (WT), *n* = 24 (KO), ^∗^*p* < 0.05, ^∗∗^*p* < 0.01, ns, not significant, two-way repeated-measures ANOVA with Holm-Sidak multiple comparison test, Student’s *t*-test [**(J)** *F*_(__5__,__125__)_ = 1.4, *p* = 0.2287; genotype, *F*_(__1__,__25__)_ = 2.409, *p* = 0.1332; time, *F*_(__5__,__125__)_ = 6.864, *p* < 0.001; **(K)** *t*_(__25__)_ = 1.434; *p* = 0.1639; **(L)** *F*_(__5__,__200__)_ = 0.315, *p* = 0.9036; genotype, *F*_(__1__,__40__)_ = 1.127, *p* = 0.2948; time, *F*_(__5__,__200__)_ = 1.348, *p* = 0.2457; **(M)** *t*_(__39__)_ = 0.6046; *p* = 0.5489; **(N)** *t*_(__24__)_ = 0.2570; *p* = 0.7994].

We first subjected *Lrrc4c^–/–^* mice to a battery of behavioral assays to determine whether NGL-1 deletion affects their behaviors. In Laboras cages, a familiar home-cage-like environment where mouse movements are monitored over several days (Quinn et al., 2003), *Lrrc4c^–/–^* mice showed strong hyperactivity during light-off periods during the last 2 days of the 3-day recordings, as measured by total distance moved (Figures 1A,B). In keeping with their horizontal hyperactivity, these mice also showed strongly increased climbing activity (Figures 1C,D). These results indicate that *Lrrc4c^–/–^* mice are hyperactive in a familiar environment.

In the open-field test, which represents a novel environment, *Lrrc4c^–/–^* mice showed moderate hyperactivity in the dark (0 lux) but not under 50-lux conditions (Figures 1E,F,J,K), suggesting that the strong hyperactivity of *Lrrc4c^–/–^* mice is dampened in a novel environment and that light further suppresses hyperactivity in a novel environment. These mice spent a normal amount of time in the center region of the open-field area (Figures 1G,H,L,M), suggesting the absence of anxiety-like behavior in this context. Immobile times were not changed (Figures 1I,N), suggesting that the normal locomotor activity observed at 50-lux light, in particular, is less likely to involve increased fear. Taken together, these results suggest that NGL-1 deletion leads to strong hyperactivity in a familiar, but not a novel, environment.

### Anxiolytic-Like Behavior and Largely Normal Depression-Like Behavior in *Lrrc4c^–/–^* Mice

**FIGURE 2:**
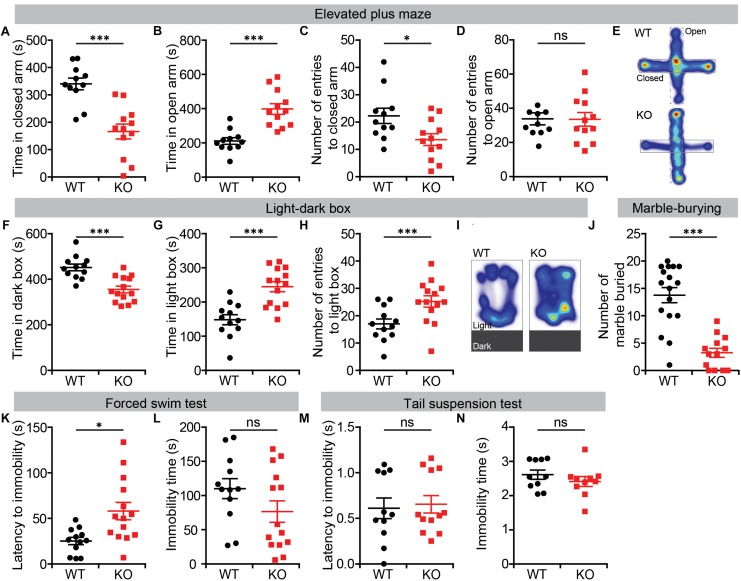
Anxiolytic-like behavior and largely normal depression-like behavior in Lrrc4c^–/–^ mice. **(A–E)** Anxiolytic-like behavior in Lrrc4c^–/–^ mice (2–5 months; male) in the EPM, as shown by time spent in closed/open arms, number of entries into closed/open arms and heat map (10 min). n = 11 (WT), n = 12 (KO), ^∗^p < 0.05, ^∗∗∗^p < 0.001, ns, not significant, Student’s t-test [**(A)** t_(__21__)_ = 4.944; p < 0.0001; **(B)** t_(__21__)_ = 5.02; p < 0.001; **(C)** t_(__8__)_ = 3.084; p = 0.0150; **(D)** t_(__21__)_ = 0.6721; p = 0.5088]. **(F–I)** Anxiolytic-like behavior in Lrrc4c^–/–^ mice (2–5 months; male) in the light–dark test, as shown by time spent in the dark/light box, number of entries into the light box, and heat map (10 min). n = 12 (WT), n = 14 (KO), ^∗∗∗^p < 0.001, Student’s t-test [**(F)** t_(__24__)_ = 4.603; p = 0.0001; **(G)** t_(__24__)_ = 4.603; p = 0.0001; **(H)** t_(__10__)_ = 6.195; p = 0.0001]. **(J)** Suppressed marble burying in Lrrc4c^–/–^ mice (2–5 months; male) in the marble-burying test (10 min). n = 17 (WT), n = 13 (KO), ^∗∗∗^p < 0.001, Student’s t-test [**(J)** t_(__28__)_ = 6.021; p < 0.001]. **(K,L)** Anti-depression–like behavior in Lrrc4c^–/–^ mice (2–5 months; male) in the forced-swim test (10 min). n = 12 (WT), n = 14 (KO), ^∗^p < 0.05, ns, not significant, Student’s t-test [**(K)** t_(__11__)_ = 2.407; p = 0.0348; **(L)** t_(__24__)_ = 1.555; p = 0.1331]. **(M,N)** Normal depression-like behavior in Lrrc4c^–/–^ mice (2–5 months; male) in the tail-suspension test (10 min). n = 11 (WT), n = 12 (KO), ns, not significant, Student’s t-test [**(M)** t_(__10__)_ = 0.5647; p = 0.5848; **(N)** t_(__18__)_ = 1.022; p = 0.3202].

We further tested *Lrrc4c^–/–^*mice for anxiety-like behaviors, first using the EPM test. In this test, *Lrrc4c^–/–^* mice spent significantly more time in open arms compared with WT mice (Figures 2A,B,E). In addition, the frequency of closed-arm entries was decreased in *Lrrc4c^–/–^* mice, although the frequency of open-arm entries was normal (Figures 2C,D), further suggestive of the anxiolytic-like behavior of *Lrrc4c^–/–^* mice.

Similar anxiolytic-like behavior was observed in the light–dark box test, where *Lrrc4c^–/–^* mice spent more time in the light chamber and less time in the dark chamber compared with WT mice (Figures 2F,G,I). *Lrrc4c^–/–^* mice also exhibited an increased frequency of light-chamber entries (Figure 2H). In addition, *Lrrc4c^–/–^* mice buried decreased numbers of marbles (Figure 2J), a potentially anxiogenic stimulus (Njung’e and Handley, 1991), further supporting an anxiolytic phenotype.

In the forced-swim test, which measures depression-like behaviors, *Lrrc4c^–/–^* mice showed increased levels of latency to immobility, but normal total immobile time (Figures 2K,L). Moreover, *Lrrc4c^–/–^* mice showed normal performance in the tail-suspension test (Figures 2M,N), indicating that these mice display largely normal depression-like behavior. These results collectively suggest that NGL-1 deletion leads to anxiolytic-like behaviors and largely normal depression-like behaviors in mice.

### Moderately Suppressed Repetitive Self-Grooming, but Normal Social Interaction and Sensorimotor Function, in *Lrrc4c^–/–^* Mice

**FIGURE 3:**
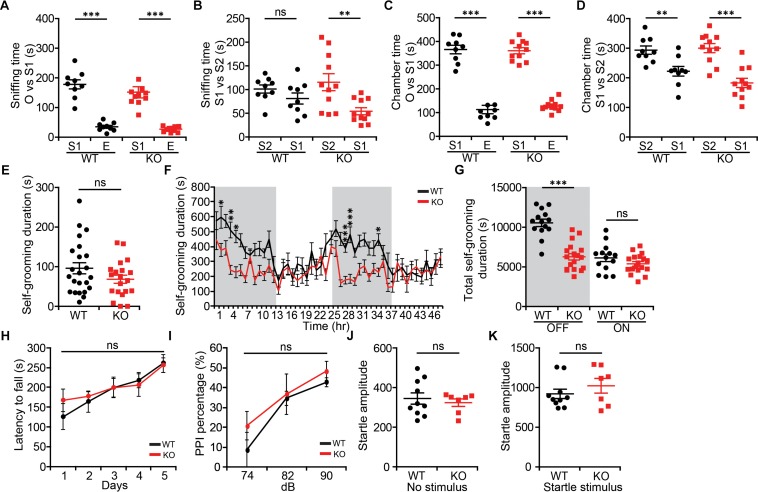
Modestly suppressed repetitive self-grooming but normal social interaction and sensorimotor function in *Lrrc4c^–/–^* mice. **(A–D)** Normal social approach behavior in *Lrrc4c^–/–^* mice (2–5 months; male) in the three-chamber test, as shown by time spent sniffing or time spent in the chamber with social (S1) targets and empty (E) chambers. Note that WT mice exhibit only a modest level of social novelty recognition, showing strongly significant recognition of a novel stranger (S2) when assessed by the time spent in chamber, but not time spent sniffing. *n* = 9 (WT), *n* = 11 (KO), ^∗∗^*p* < 0.01, ^∗∗∗^*p* < 0.001, ns, not significant, two-way repeated-measures ANOVA with Holm-Sidak multiple comparison test [**(A)** interaction, *F*_(__1__,__36__)_ = 0.128, *p* = 0.7226; genotype, *F*_(__1__,__36__)_ = 1.087, *p* = 0.3042; subject, *F*_(__1__,__36__)_ = 141.2, *p* < 0.001; **(B)** interaction, *F*_(__1__,__36__)_ = 2.691, *p* = 0.1096; genotype, *F*_(__1__,__36__)_ = 0.2517, *p* = 0.6189; subject, *F*_(__1__,__36__)_ = 10.48, *p* = 0.0026; **(C)** interaction, *F*_(__1__,__36__)_ = 0.07257, *p* = 0.7892; genotype, *F*_(__1__,__36__)_ = 0.0068, *p* = 0.9349; subject, *F*_(__1__,__36__)_ = 289.2, *p* < 0.001; **(D)** interaction, *F*_(__1__,__36__)_ = 2.196, *p* = 0.1471; genotype, *F*_(__1__,__36__)_ = 1.116, *p* = 0.2977; subject, *F*_(__1__,__36__)_ = 36.93, *p* < 0.001]. **(E)** Normal self-grooming in *Lrrc4c^–/–^* mice (2–5 months; male) in novel bedded home cages, as shown by total self-grooming duration (10 min). *n* = 23 (WT), *n* = 20 (KO), ns, not significant, Student’s *t*-test [**(E)** *t*_(__41__)_ = 1.559; *p* = 0.1266]. **(F,G)** Suppressed self-grooming in *Lrrc4c^–/–^* mice (2–5 months; male) in Laboras cages, as shown by time spent in self-grooming (2 days). *n* = 23 (WT), *n* = 20 (KO), ^∗^*p* < 0.05, ^∗∗^*p* < 0.01, ^∗∗∗^*p* < 0.001, ns, not significant, two-way repeated-measures ANOVA with Holm-Sidak multiple comparison test, Student’s *t*-test [**(F)** interaction, *F*_(47,1410)_ = 2.429, *p* < 0.0001; time, *F*_(47,1410)_ = 6.467, *p* < 0.0001; genotype, *F*_(1,30)_ = 26.73, *p* < 0.0001; 2 h, *p* = 0.0499; 4 h, *p* = 0.0032; 5 h, *p* = 0.0143; 27 h, *p* = 0.0005; 29 h, *p* = 0.0005; 35 h, *p* = 0.0499; **(G)** interaction, *F*_(__1__,__60__)_ = 19.14, *p* < 0.001; genotype, *F*_(__1__,__60__)_ = 38.99, *p* < 0.001; time, *F*_(__1__,__60__)_ = 43.19, *p* < 0.001; OFF, *p* = 0.1913, ON, *p* < 0.001]. **(H)** Normal motor coordination and learning in *Lrrc4c^–/–^* mice (2–5 months; male) in the rotarod test. *n* = 7 (WT), *n* = 9 (KO), ns, not significant, two-way repeated-measures ANOVA with Holm-Sidak multiple comparison test [**(H)** interaction, *F*_(__4__,__56__)_ = 0.4294, *p* = 0.7867; genotype, *F*_(__1__,__14__)_ = 0.163, *p* = 0.6925; time, *F*_(__4__,__56__)_ = 7.028, *p* = 0.0001]. **(I–K)** Normal pre-pulse inhibition **(I)** and startle responses **(J,K)** in *Lrrc4c^–/–^* mice (2–5 months; male). *n* = 10 (WT), *n* = 7 (KO), ns, not significant, two-way repeated-measures ANOVA with Holm-Sidak multiple comparison test, Student’s *t*-test [**(J)** *t*_(__15__)_ = 0.553; *p* = 0.5884; **(K)** *t*_(__15__)_ = 0.9855; *p* = 0.3400].

Because NGL-1/LRRC4C (Walker and Scherer, 2013; Maussion et al., 2017) and the NGL-1-interacting proteins netrin-G1 and CDKL5 (O’Roak et al., 2012; Posar et al., 2015; Zhou et al., 2017; Zhu and Xiong, 2019) have been associated with ASD, we also examined social and repetitive behaviors in *Lrrc4c^–/–^* mice. In the three-chamber test (Moy et al., 2004; Silverman et al., 2010), social approach of *Lrrc4c^–/–^* mice was comparable to that of WT mice, as shown by sniffing and chamber time (Figures 3A,C). Social-novelty recognition was also normal in *Lrrc4c^–/–^* mice (Figures 3B,D), although WT mice showed a partial failure of social novelty recognition.

In tests measuring repetitive behaviors, *Lrrc4c^–/–^* mice showed normal self-grooming levels in a bedded novel home cage (Figure 3E). In Laboras cages, however, *Lrrc4c^–/–^* mice showed suppressed self-grooming (Figures 3F,G). This discrepancy might be attributable to the difference between novel and familiar environments.

In addition to these two behavioral domains, we also examined sensory and motor functions of *Lrrc4c^–/–^* mice using rotarod and pre-pulse inhibition tests. We found no significant differences between genotypes in either assay (Figures 3H–K), suggesting that these mice have normal levels of motor coordination and motor learning, as well as sensorimotor gating (Geyer et al., 2001). These results collectively suggest that NGL-1 deletion suppresses repetitive self-grooming in a familiar environment, but has no effect on social or sensory motor functions.

### Suppressed Working and Contextual Memory in *Lrrc4c^–/–^* Mice

**FIGURE 4:**
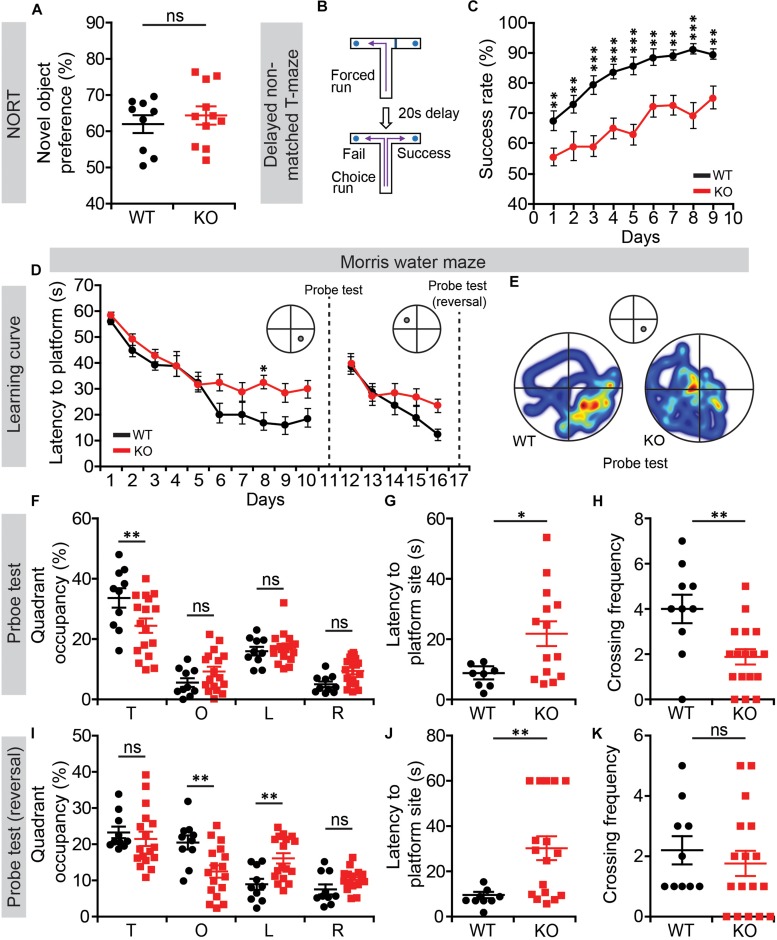
Suppressed working and contextual memory in *Lrrc4c^–/–^* mice. **(A)** Normal novel-object recognition in *Lrrc4c^–/–^* mice (2–5 months; male). *n* = 9 (WT), *n* = 11 (KO), ns, not significant, Student’s *t*-test [*t*_(__18__)_ = 0.6718; *p* = 0.5102]. **(B,C)** Suppressed working memory in *Lrrc4c^–/–^* mice (2–5 months; male) in the delayed non-match choice T-maze test. *n* = 29 (WT), *n* = 22 (KO), ^∗∗^*p* < 0.01, ^∗∗∗^*p* < 0.001, two-way repeated-measures ANOVA with Holm-Sidak multiple comparison test [**(C)** interaction, *F*_(__8__,__392__)_ = 1.01, *p* = 0.4276; genotype, *F*_(__1__,__49__)_ = 44.74, *p* < 0.001; time, *F*_(__8__,__392__)_ = 15.88, *p* < 0.001; day 1, *p* = 0.0059, day 2, *p* = 0.0033, day 3, *p* < 0.001, day 4, *p* = 0.0001, day 5, *p* < 0.001, day 6, *p* = 0.0010, day 7, *p* = 0.0008, day 8, *p* < 0.001, day 9, *p* = 0.0032]. **(D–K)** Suppressed spatial learning and memory in *Lrrc4c^–/–^* mice (2–5 months; male) in the Morris water-maze test, as shown by performance in initial learning **(D)**, initial probe **(E–H)**, reversal learning **(D)**, and reversal probe **(I–K)** phases. *n* = 10 (WT), *n* = 17 (KO), ^∗^*p* < 0.05, ^∗∗^*p* < 0.01, ns, not significant, two-way repeated-measures ANOVA with Holm-Sidak multiple comparison test, Student’s *t*-test [**(D)** interaction, *F*_(__9__,__225__)_ = 1.65, *p* = 0.1025; genotype, *F*_(__1__,__25__)_ = 6.576, *p* = 0.0167; time, *F*_(__9__,__225__)_ = 27.25, *p* < 0.001; day 8, *p* = 0.0215; **(F)** interaction, *F*_(__3__,__75__)_ = 4.298, *p* = 0.0075; genotype, *F*_(__1__,__25__)_ = 1.735, *p* = 0.1997; time, *F*_(__3__,__75__)_ = 45.99, *p* < 0.001; target, *p* = 0.0028; **(G)** *t*_(__20__)_ = 2.525; *p* = 0.0201; **(H)** *t*_(__25__)_ = 3.227; *p* = 0.0035; **(I)** interaction, *F*_(__3__,__75__)_ = 5.631, *p* = 0.0016; genotype, *F*_(__1__,__25__)_ = 0.7606, *p* = 0.3914; time, *F*_(__3__,__75__)_ = 18.15, *p* < 0.001; opposite, *p* = 0.0040 left, *p* = 0.0091; **(J)** *t*_(__24__)_ = 2.966; *p* = 0.0067; **(K)** *t*_(__25__)_ = 0.6689; *p* = 0.5097].

We next subjected *Lrrc4c^–/–^* mice to tests of learning and memory. In the novel-object-recognition test, *Lrrc4c^–/–^* mice showed normal levels of novel-object preference (Figure 4A), indicative of normal object memory. In the T-maze test involving a delayed, non-matched choice, *Lrrc4c^–/–^* mice showed strongly decreased success rates from the start and across all test days (Figures 4B,C), suggestive of working memory impairments.

In the Morris water maze test, *Lrrc4c^–/–^* mice performed poorly during learning, probe, and reversal phases (Figures 4D–K), indicative of suppressed spatial learning and memory. These results collectively suggest that *Lrrc4c^–/–^* mice have impaired working and spatial memory, but normal object-recognition memory functions. This is in line with the impaired short-term plasticity in the TA pathway, known to be associated with working memory (Yamamoto et al., 2014), in the hippocampus of *Lrrc4c^–/–^* mice.

### Delays in Perceptual Learning With Normal Visual Attention in *Lrrc4c^–/–^* Mice

**FIGURE 5:**
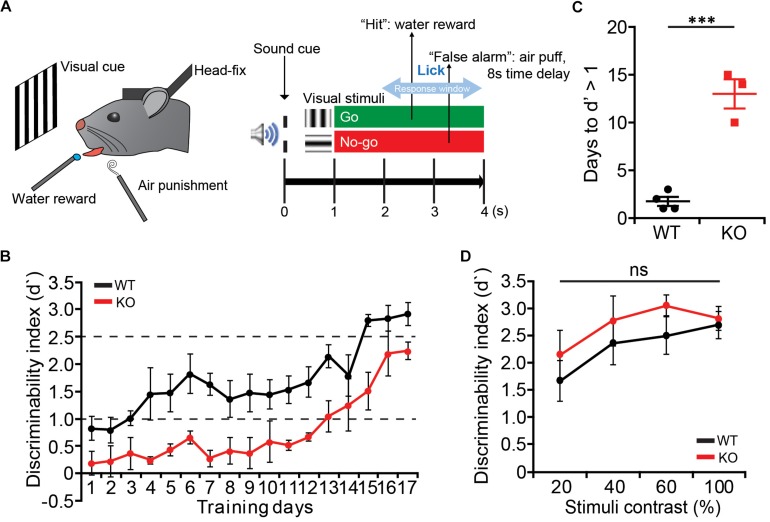
Delayed learning of visual discrimination but normal visual attention in *Lrrc4c^–/–^* mice. **(A)** Schematic illustration of the visual discrimination task for head-fixed mice. (Left) Schematic of mice under the task. (Right) Task design. **(B)** The learning curve of WT (black) and KO mice (red). Note that *Lrrc4c^–/–^* mice show slower learning of the task. *n* = 4 (WT), *n* = 3 (KO). **(C)** Learning days required for mice to reach the initial learning stage (d′ = 1). *n* = 4 (WT), *n* = 3 (KO), ^∗∗∗^*p* < 0.001, Student’s *t*-test. **(D)** Discrimination performance of mice at lower contrasts of visual stimuli. Note that the *Lrrc4c^–/–^* mice show similar levels of discrimination performance compared to the WT mice at low-contrast visual stimuli. *n* = 4 (WT), *n* = 3 (KO), ns, not significant, two-way ANOVA [**(B)** *t*_(__5__)_ = 8.048; *p* = 0.0005; **(D)** interaction, *F*_(__3__,__15__)_ = 1.032, *p* = 0.4066; genotype, *F*_(__1__,__5__)_ = 0.6932, *p* = 0.4430; stimulus, *F*_(__3__,__15__)_ = 18.09, *p* < 0.0001].

We next tested whether *Lrrc4c^–/–^* mice have any deficits in attention. To dissociate the perceptual learning and attention, we trained mice to perform an orientation discrimination task under the head-restrained condition (Lee et al., 2012; Pinto et al., 2013; Zhang et al., 2016a) (Figure 5A). *Lrrc4c^–/–^* mice showed delayed learning compared with WT mice, showing longer days of training to reach the similar level of discrimination performance (Figures 5B,C). Although it took longer for *Lrrc4c^–/–^* mice to learn the task, we continued to train both WT and *Lrrc4c^–/–^* mice until they became experts in performing visual discrimination task (d′ > 2.5). We then lowered the contrast of visual stimuli (100, 80, 60, and 20%) to test the level of visual attention in learned mice. We found that *Lrrc4c^–/–^* mice show a similar level of discrimination compared with WT mice even at the lowest contrast (Figure 5D), suggesting that *Lrrc4c^–/–^* mice have normal levels of visual attention.

### Suppressed Neuronal Activity Under Baseline and Anxiety-Inducing Conditions in *Lrrc4c^–/–^* Mice

To identify brain regions associated with the anxiolytic-like behavior of *Lrrc4c^–/–^* mice, we examined the levels of c-fos, a marker of neuronal activity (Sheng and Greenberg, 1990), under baseline conditions and in the EPM test.

**FIGURE 6:**
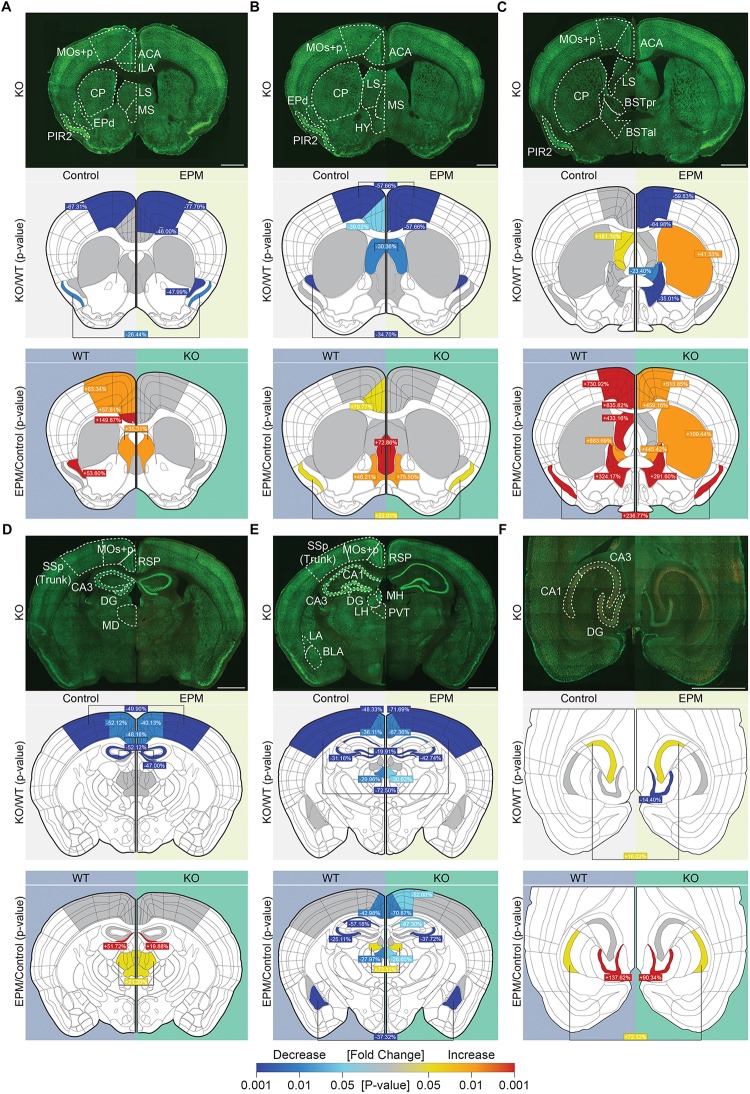
Suppressed neuronal activity under baseline and anxiety-inducing EPM conditions in *Lrrc4c^–/–^* mice. **(A–F)** Representative coronal and horizontal brain sections from *Lrrc4c^–/–^* mice (3–4 months, male; row 1 of each figure panel) at six positions: **(A–E)** coronal sections along the rostral-caudal axis; **(F)** horizontal sections showing the ventral hippocampus. Brain slices were stained for c-fos (red) and the neuronal marker NeuN (green) under baseline (control; left) and EPM (right) conditions. Examples of WT brains are not shown owing to space limitations. Graphical representations of each region were used to display statistically significant changes (*p*-values indicated by the colored scale bar at the bottom) between different genotypes under baseline or EPM conditions (row 2 of each panel) and significant changes between baseline and EPM conditions within the same genotype (row 3 of each panel). In addition, percent changes for each region, calculated as (KO – WT)/WT x 100, are indicated for the comparison of mean values. Cases where values are written in the middle and linked to both sides indicate that only one of the two parameters (genotype or condition) is statistically significant, but there is no interaction between parameters. ACC, anterior cingulate area or ACA cortex; BLA, basolateral amygdala; BNSTal, anterolateral bed nuclei of the stria terminalis; BNSTpr, posterior bed nuclei of the stria terminalis; CA1, *cornu* ammonus 1; CA3, *cornu* ammonus 3; CP, caudoputamen; DG, dentate gyrus; EPd, dorsal endopiriform nucleus; HY, hypothalamus; ILA, infralimbic area; LA, lateral amygdala; LH, lateral habenula; LS, lateral septum; MD, mediodorsal nucleus of the thalamus; MOs + p, secondary and primary motor area; MS, medial septal nucleus; PIR2, pyramidal layer of piriform area; PVT, paraventricular nucleus of the thalamus; RSP, retrosplenial area; SSp, primary somatosensory area. *n* = 16 slices from 4 mice (WT), 16, 4 (KO), two-way ANOVA (genotype × stimulus) with Tukey’s HSD test. Scale bar: 1 mm.

Under baseline conditions, c-fos signals in *Lrrc4c^–/–^* brains were substantially decreased relative to those in WT mice in multiple cortical and subcortical regions, including the ACC, motor cortex (MO), piriform cortex (PIR2), endopiriform nucleus (EPd), retrosplenial area (RSP), SSp (primary somatosensory area), paraventricular nucleus of the thalamus (PVT), and dorsal (d) regions of the hippocampus (dDG, dCA3, and dCA1), but not ventral (v) regions (Figures 6A–F; second rows). Only two regions—the lateral septum (LS; in section 3, but not 2) and vCA3—showed modestly increased c-fos levels under baseline conditions (Figures 6B,C,F and Supplementary Figure S1).

Interestingly, exposure of *Lrrc4c^–/–^* mice to an anxiety-inducing stimulus (EPM test) revealed similarly lower c-fos signals (relative to WT levels) across multiple brain regions, including the ACC, MO, PIR2, EPd, LS, bed nuclei of the stria terminalis (BNST), RSP, SSp, PVT, and hippocampus (dDG, dCA3, dCA1, and vDG) (Figure 6). Only two regions—the caudoputamen (CP) and vCA3—showed higher c-fos levels in *Lrrc4c^–/–^* mice relative to WT mice upon exposure to the EPM stimulus (Figure 6C).

To better understand these changes, we also compared c-fos levels between baseline and EPM conditions within the same genotype (WT or KO). In WT mice administered EPM tests, c-fos levels increased in multiple brain regions, including the ACC, ILA, MO, PIR2, EPd, LS, medial septum (MS), BNST, mediodorsal nucleus of the thalamus (MD), lateral habenula (LH), and hippocampus (dDG [section 4], vDG, and vCA1) (Figure 6). A few WT brain regions, including the RSP, PVT, dCA3, dCA1 and basolateral amygdala (BLA), showed decreased c-fos levels upon exposure to the EPM stimulus compared with baseline conditions (Figure 6).

*Lrrc4c^–/–^* brain regions displayed c-fos levels distinct from those in WT brains under EPM conditions. Certain brain regions that showed strong EPM-induced increases in c-fos levels in WT mice, including the ACC, ILA, MO, EPd, and LS (section 3, but not section 1 or 2), failed to show such increases in *Lrrc4c^–/–^* mice. Notably, the CP (in the dorsal striatum; section 3, but not section 1 or 2) in the *Lrrc4c^–/–^* brain showed EPM-induced increases in c-fos levels that were greater than those in WT mice (Figures 5A–C).

Collectively, these results suggest that neuronal activity is decreased in various *Lrrc4c^–/–^* brain regions under baseline conditions, and that EPM stimulation does not increase neuronal activity in *Lrrc4c^–/–^* brain regions as strongly as it does in corresponding regions of the WT brain. These decreases in neuronal activity under baseline and EPM conditions might contribute to anxiolytic-like behaviors in *Lrrc4c^–/–^* mice.

### *Lrrc4c^–/–^* Brain Regions With Distinct Changes in Neuronal Activity Upon EPM Stimulation

**FIGURE 7:**
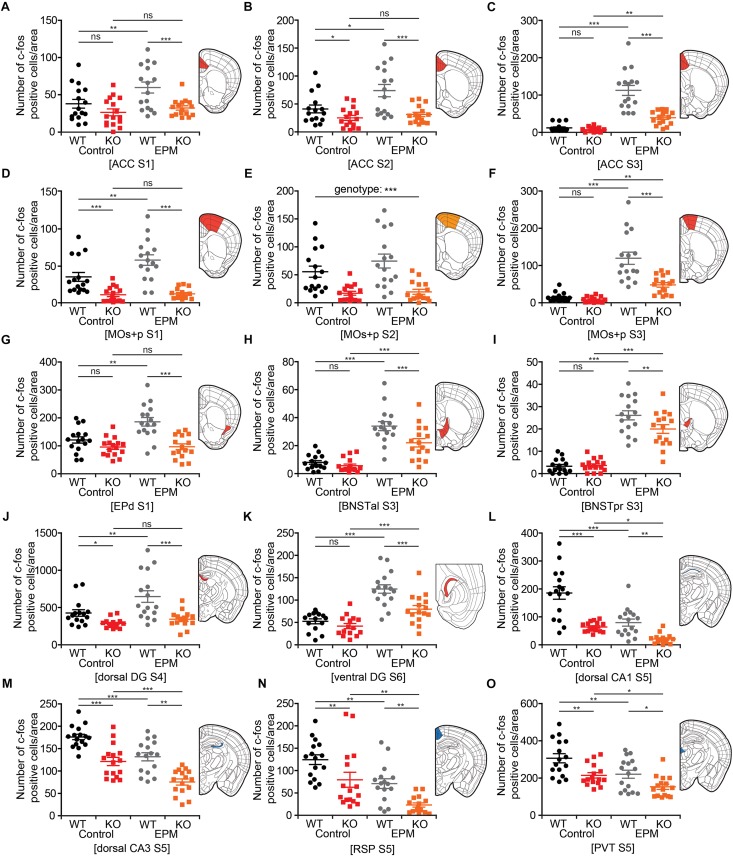
*Lrrc4c^–/–^* brain regions with distinct changes in neuronal activity upon EPM stimulation. **(A–K)** Brain regions at examined brain sections (S) that show strong EPM-induced increases in c-fos levels (indicated by the number of c-fos-positive cells) in WT mice, but moderate or no increases in *Lrrc4c^–/–^* mice (3–4 months; male). Results for the colored regions within each brain diagram are indicated in the corresponding color-coded bar graphs. Blue and red shades in the brain diagrams indicate the statistical significance of both main factors or their interaction. Orange shades indicate statistical significance of only the genotype factor. *n* = 16 slices from 4 mice (WT), *n* = 16 (4) (KO), ^∗^*p* < 0.05, ^∗∗^*p* < 0.01, ^∗∗∗^*p* < 0.001, ns, not significant, two-way ANOVA (genotype × stimulus) with Holm-Sidak multiple comparison test [**(A)** interaction, *F*_(__1__,__63__)_ = 2.14, *p* = 0.149; genotype, *F*_(__1__,__63__)_ = 12.79, *p* = 0.0007; stimulus, *F*_(__1__,__63__)_ = 6.51, *p* = 0.0133; WT (E vs. C), *p* = 0.0062, KO (E vs. C), *p* = 0.4444, Control (WT vs. KO), *p* = 0.1401, EPM (WT vs. KO), *p* = 0.0007; **(B)** two-way ANOVA; interaction, *F*_(__1__,__63__)_ = 1.99, *p* = 0.1631; genotype, *F*_(__1__,__63__)_ = 18.11, *p* < 0.001; stimulus, *F*_(__1__,__63__)_ = 5.01, *p* = 0.0289; WT (E vs. C), *p* = 0.0123, KO (E vs. C), *p* = 0.5608, Control (WT vs. KO), *p* = 0.0489, EPM (WT vs. KO), *p* = 0.0002; **(C)** two-way ANOVA; interaction, *F*_(__1__,__63__)_ = 22.42, *p* < 0.001; genotype, *F*_(__1__,__63__)_ = 29.45, *p* < 0.001; stimulus, *F*_(__1__,__63__)_ = 85.22, *p* < 0.001; WT (E vs. C), *p* < 0.001, KO (E vs. C), *p* = 0.0023, Control (WT vs. KO), *p* = 0.6266, EPM (WT vs. KO), *p* < 0.001; **(D)** two-way ANOVA; interaction, *F*_(__1__,__63__)_ = 4.82, *p* = 0.0321; genotype, *F*_(__1__,__63__)_ = 50.97, *p* < 0.001; stimulus, *F*_(__1__,__63__)_ = 6.04, *p* = 0.0169; WT (E vs. C), *p* = 0.0017, KO (E vs. C), *p* = 0.8527, Control (WT vs. KO), *p* = 0.0009, EPM (WT vs. KO), *p* < 0.001; **(E)** interaction, *F*_(__1__,__60__)_ = 0.9534, *p* = 0.3328; genotype, *F*_(__1__,__60__)_ = 30.7815, *p* < 0.001; stimulus, *F*_(__1__,__60__)_ = 1.637, *p* = 0.2057; **(F)** interaction, *F*_(__1__,__63__)_ = 13.27, *p* = 0.0006; genotype, *F*_(__1__,__63__)_ = 19.17, *p* < 0.001; stimulus, *F*_(__1__,__63__)_ = 66.44, *p* < 0.001; WT (E vs. C), *p* < 0.001, KO (E vs. C), *p* = 0.0023, Control (WT vs. KO), *p* = 0.6046, EPM (WT vs. KO), *p* < 0.001; **(G)** interaction, *F*_(__1__,__63__)_ = 7.67, *p* = 0.0074; genotype, *F*_(__1__,__63__)_ = 12.79, *p* < 0.001; stimulus, *F*_(__1__,__63__)_ = 6.51, *p* = 0.0059; WT (E vs. C), *p* = 0.0002, KO (E vs. C), *p* = 0.4444, Control (WT vs. KO), *p* = 0.1259, EPM (WT vs. KO), *p* < 0.001; **(H)** interaction, *F*_(__1__,__63__)_ = 4.02, *p* = 0.0494; genotype, *F*_(__1__,__63__)_ = 9.03, *p* = 0.0039; stimulus, *F*_(__1__,__63__)_ = 79.76, *p* < 0.001; WT (E vs. C), *p* < 0.001, KO (E vs. C), *p* < 0.001, Control (WT vs. KO), *p* = 0.4828, EPM (WT vs. KO), *p* = 0.0008; **(I)** interaction, *F*_(__1__,__63__)_ = 4.53, *p* = 0.0373; genotype, *F*_(__1__,__63__)_ = 3.64, *p* = 0.0612; stimulus, *F*_(__1__,__63__)_ = 167.04, *p* < 0.001; WT (E vs. C), *p* < 0.001, KO (E vs. C), *p* < 0.001, Control (WT vs. KO), *p* = 0.876, EPM (WT vs. KO), *p* = 0.0059; **(J)** interaction, *F*_(__1__,__63__)_ = 5.32, *p* = 0.0245; genotype, *F*_(__1__,__63__)_ = 9.4, *p* = 0.0032; stimulus, *F*_(__1__,__63__)_ = 127.03, *p* < 0.001; WT (E vs. C), *p* = 0.0018, KO (E vs. C), *p* = 0.3961, Control (WT vs. KO), *p* = 0.0422, EPM (WT vs. KO), *p* < 0.001; **(K)** interaction, *F*_(__1__,__58__)_ = 5.2833, *p* = 0.0252; genotype, *F*_(__1__,__58__)_ = 53.793, *p* < 0.001; stimulus, *F*_(__1__,__58__)_ = 13.8661, *p* = 0.0004; WT (E vs. C), *p* = 0.0001, KO (E vs. C), *p* = 0.0009, Control (WT vs. KO), *p* = 0.3255, EPM (WT vs. KO), *p* = 0.0002]. **(L–O)** Brain regions that show strong EPM-induced decreases (not increases) in c-fos levels in both WT and *Lrrc4c^–/–^* mice (3–4 months; male). Note that because baseline c-fos levels are lower in *Lrrc4c^–/–^* mice, final c-fos levels after EPM stimulation are also lower in *Lrrc4c^–/–^* mice. *n* = 16 (4) (WT), *n* = 16 (4) (KO), ^∗^*p* < 0.05, ^∗∗^*p* < 0.01, ^∗∗∗^*p* < 0.001, two-way ANOVA (genotype × stimulus) Holm-Sidak multiple comparison test [**(L)** interaction, *F*_(__1__,__63__)_ = 5.78, *p* = 0.0193; genotype, *F*_(__1__,__63__)_ = 47.13, *p* < 0.001; stimulus, *F*_(__1__,__63__)_ = 32.18, *p* < 0.001; WT (E vs. C), *p* < 0.001, KO (E vs. C), *p* = 0.0243, Control (WT vs. KO), *p* < 0.001, EPM (WT vs. KO), *p* = 0.0025; **(M)** interaction, *F*_(__1__,__63__)_ = 0.01, *p* = 0.9247; genotype, *F*_(__1__,__63__)_ = 49.42, *p* < 0.001; stimulus, *F*_(__1__,__63__)_ = 32.32, *p* < 0.001; WT (E vs. C), *p* = 0.0003, KO (E vs. C), *p* = 0.0002, Control (WT vs. KO), *p* = 0.0001, EPM (WT vs. KO), *p* = 0.0001; **(N)** interaction, *F*_(__1__,__63__)_ = 0.02, *p* = 0.9018; genotype, *F*_(__1__,__63__)_ = 16.02, *p* = 0.0002; stimulus, *F*_(__1__,__63__)_ = 22.47, *p* < 0.001; WT (E vs. C), *p* = 0.0018, KO (E vs. C), *p* = 0.0011, Control (WT vs. KO), *p* = 0.008, EPM (WT vs. KO), *p* = 0.005; **(O)** interaction, *F*_(__1__,__63__)_ = 0.4, *p* = 0.5273; genotype, *F*_(__1__,__63__)_ = 17.49, *p* < 0.001; stimulus, *F*_(__1__,__63__)_ = 14.92, *p* = 0.0003; WT (E vs. C), *p* = 0.0023, KO (E vs. C), *p* = 0.0261, Control (WT vs. KO), *p* = 0.0012, EPM (WT vs. KO), *p* = 0.0149].

To obtain a clearer picture of EPM-induced changes in neuronal activity in the brains of WT and *Lrrc4c^–/–^* mice, we highlighted brain regions (in colored-coded bar graphs) and their corresponding c-fos levels under baseline and EPM conditions. We found that c-fos levels in the ACC were similar or lower in *Lrrc4c^–/–^* mice relative to those in WT mice under baseline conditions (Figures 7A–C). Upon EPM stimulation, c-fos activity in *Lrrc4c^–/–^* mice was not as strongly increased as in WT mice (Figures 7A–C), further enhancing the difference in c-fos levels between WT and *Lrrc4c^–/–^* mice. Similar differences in baseline c-fos levels and EPM-induced changes in c-fos levels were observed in other brain regions, including the MO, BNST, EPd, and DG (Figures 7D–K).

Intriguingly, some other brain regions showed EPM-induced decreases in c-fos levels upon EPM stimulation in both WT and *Lrrc4c^–/–^* mice; these included the dCA1, dCA3, RSP and PVT (Figures 7L–O). Because baseline c-fos levels in these regions were lower in *Lrrc4c^–/–^* mice, final c-fos levels after EPM stimulation were still lower in *Lrrc4c^–/–^* mice relative to those in WT mice (Figures 7L–O). Notably, although the direction of EPM-induced changes in c-fos levels in this group of brain regions (CA1, CA3, RSP, PVT) was opposite that of the regions mentioned above (ACC, MO, DG, BNST, and EPd), the end result—lower c-fos activity in *Lrrc4c^–/–^* regions under EPM conditions—seemed to be similar. Interestingly, while exposure to the EPM stimulus caused a decrease in c-fos expression, no genotype difference was observed in the BLA, a well-known anxiety-related brain region (Tye et al., 2011; Felix-Ortiz et al., 2013).

These results collectively suggest that NGL-1 deletion leads to distinct or opposite changes in neuronal activity under EPM conditions in two groups of *Lrrc4c^–/–^* brain regions; however, this seems to culminate in lower c-fos activity in the majority of brain regions under EPM conditions.

### Altered Synaptic Transmission and Intrinsic Excitability in *Lrrc4c^–/–^* Brain Regions

The above-mentioned differences in the activity of *Lrrc4c^–/–^* neurons in different brain areas under baseline and EPM conditions may reflect changes in synaptic transmission or neuronal excitability. To test this, we measured these parameters in ACC, MO, and vDG regions of *Lrrc4c^–/–^* brain slices. These regions were chosen because of the literature that links activities of these regions with anxiety (Wassermann et al., 2001; Kim et al., 2011; Kheirbek et al., 2013) and the strong expression of NGL-1 (Choi et al., 2019).

Intriguingly, layer 2/3 pyramidal neurons in the *Lrrc4c^–/–^* ACC (section 2) showed increased frequency, but normal amplitude, of spontaneous excitatory post-synaptic currents (sEPSCs), whereas neither the frequency nor amplitude of spontaneous inhibitory post-synaptic currents (sIPSCs) was affected (Figures 8A,B). Neuronal excitability of these neurons was also largely unaffected, as shown by input resistance and current-firing curve, although there was a moderate decrease in resting membrane potential (Figures 8C–F). Therefore, the output functions of these neurons are likely to be increased under baseline and EPM conditions. This prediction, however, appears to contrast with the lower c-fos signals observed in the *Lrrc4c^–/–^* ACC under baseline and EPM conditions in mice (Figure 7B).

**FIGURE 8:** Altered synaptic transmission and intrinsic excitability in the *Lrrc4c^–/–^* ACC and MO. **(A,B)** Increased frequency (but not amplitude) of sEPSCs, and normal sIPSCs in layer 2/3 pyramidal neurons in the ACC of *Lrrc4c^–/–^* mice (2–4 months). *n* = 14 neurons (3 mice) (WT), *n* = 13 (3) (KO), ^∗∗∗^*p* < 0.001, ns, not significant, Student’s *t*-test [**(A)** sEPSC amplitude: *t*_(__25__)_ = 1.277, *p* = 0.2133; sEPSC frequency: *t*_(__25__)_ = 4.767, *p* = 0.0001; **(B)** sIPSC amplitude: *t*_(__32__)_ = 1.717, *p* = 0.0956; sIPSC frequency: *t*_(__31__)_ = 0.1636, *p* = 0.8711]. **(C–F)** Largely normal intrinsic neuronal activity in layer 2/3 pyramidal neurons in the ACC *of Lrrc4c^–/–^* mice (2–4 months), as shown by modestly decreased resting membrane potential, but normal input resistance and current-firing curve. *n* = 16 (3) (WT), *n* = 23 (4) (KO), ^∗^*p* < 0.05, ns, not significant, Student’s *t*-test, two-way repeated measures ANOVA with Holm-Sidak multiple comparison test [**(C)** *t*_(__37__)_ = 2.178, *p* = 0.0359; **(D)** *t*_(__37__)_ = 1.295, *p* = 0.2034; **(E)** interaction, *F*_(__17__,__629__)_ = 2.601, *p* = 0.0004; genotype, *F*_(__1__,__37__)_ = 1.773, *p* = 0.1912; current, *F*_(__17__,__629__)_ = 436.1, *p* < 0.0001; **(F)** interaction, *F*_(__9__,__333__)_ = 1.512, *p* = 0.1420; genotype, *F*_(__1__,__37__)_ = 2.249, *p* = 0.1422; current, *F*_(__9__,__333__)_ = 209.9, *p* < 0.0001]. **(G,H)** Normal sEPSCs and sIPSCs in layer 2/3 pyramidal neurons in the MO of *Lrrc4c^–/–^* mice (2–4 months). *n* = 20 (4) (WT), *n* = 14 (3) (KO), ns, not significant, Student’s *t*-test [**(G)** sEPSC amplitude: *t*_(__32__)_ = 0.3825, *p* = 0.7046; sEPSC frequency: *t*_(__31__)_ = 0.6255, *p* = 0.5362; (**H**) sIPSC amplitude: *t*_(__30__)_ = 1.917, *p* = 0.0648; sIPSC frequency: *t*_(__30__)_ = 1.382, *p* = 0.1772]. **(I–L)** Decreased intrinsic neuronal activity in layer 2/3 pyramidal neurons in the MO of *Lrrc4c^–/–^* mice (2–4 months), as shown by decreased current-firing curve, although resting membrane potential and input resistance are normal. *n* = 20 (4) (WT), *n* = 21 (4) (KO), ^∗^*p* < 0.05, ns, not significant, Student’s *t*-test, two-way repeated measures ANOVA with Holm-Sidak multiple comparison test [**(I)** *t*_(__44__)_ = 0.8966, *p* = 0.3748; **(J)** *t*_(__44__)_ = 1.066, *p* = 0.2924; **(K)** interaction, *F*_(__17__,__663__)_ = 1.553, *p* = 0.0713; genotype, *F*_(__1__,__39__)_ = 0.0918, *p* = 0.7636; current, *F*_(__17__,__663__)_ = 926.1, *p* < 0.0001; **(L)** interaction, *F*_(__14__,__518__)_ = 3.254, *p* < 0.0001; genotype, *F*_(__1__,__37__)_ = 3.114, *p* = 0.0859; current, *F*_(__14__,__518__)_ = 83.52, *p* < 0.0001; 330 pA, *p* = 0.0340, 360 pA, *p* = 0.0126, 390 pA, *p* = 0.0217, 420 pA, *p* = 0.0425].

In layer 2/3 pyramidal neurons of the *Lrrc4c^–/–^* MO (section 2), both sEPSCs and sIPSCs were normal (Figures 8G,H). In contrast, neuronal excitability of these neurons was decreased, as shown by the current-firing curve, although input resistance and resting membrane potential were unaltered (Figures 8I–L). These results are consistent with the lower c-fos signals observed in the *Lrrc4c^–/–^* MO under baseline and EPM conditions (Figure 7E).

**FIGURE 9:**
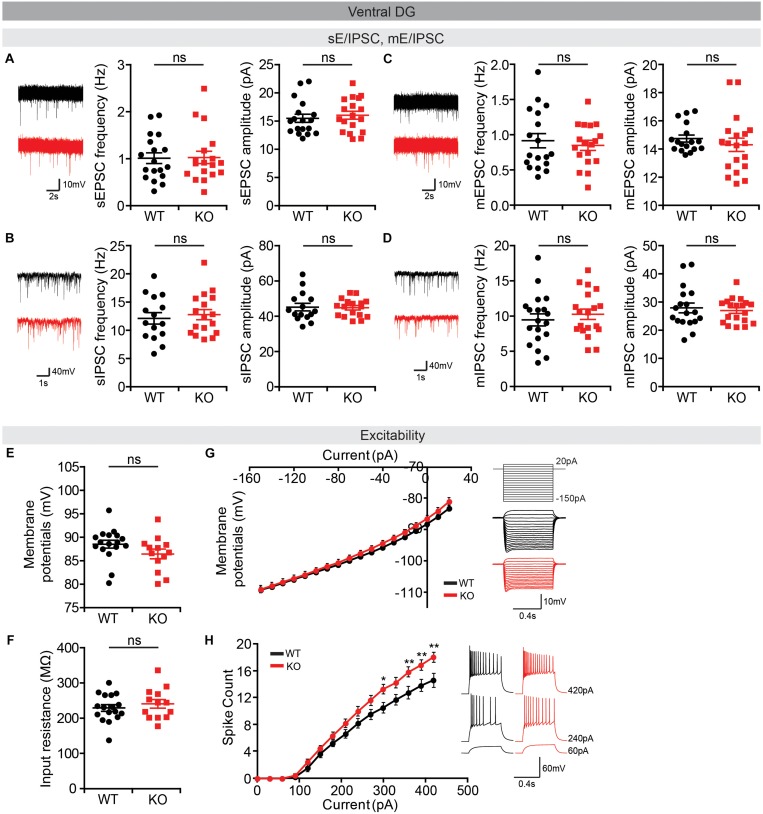
Normal synaptic transmission but increased intrinsic excitability in *Lrrc4c^–/–^* vDG neurons. **(A,B)** Normal sEPSCs and sIPSCs in vDG neurons in the hippocampus of *Lrrc4c^–/–^* mice (2–4 months). *n* = 18 (3) (WT), *n* = 18 (3) (KO) (sEPSC), *n* = 15 (3) (WT), *n* = 17 (3) (KO) (sIPSC), ns, not significant, Student’s *t*-test **[(A)** sEPSC amplitude: *t*_(__34__)_ = 0.5486, *p* = 0.5869; sEPSC frequency: *t*_(__34__)_ = 0.0886, *p* = 0.9299; **(B)** sIPSC amplitude: *t*_(__30__)_ = 0.1498, *p* = 0.8819; sIPSC frequency: *t*_(__29__)_ = 0.7358, *p* = 0.4677]. **(C,D)** Normal mEPSCs and mIPSCs in vDG neurons in the hippocampus of *Lrrc4c^–/–^* mice (2–4 months). *n* = 18 (4) (WT), *n* = 19 (4) (KO) (mEPSC), *n* = 18 (3) (WT), *n* = 19 (3) (KO) (mIPSC), ns, not significant, Mann–Whitney test, Student’s *t*-test **[(A)** mEPSC amplitude: *U* = 130.5, *p* = 0.3340; mEPSC frequency: *t*_(__34__)_ = 0.5379, *p* = 0.5942; **(B)** mIPSC amplitude: *U* = 166.5, *p* = 0.8987; mIPSC frequency: *t*_(__36__)_ = 0.7231, *p* = 0.4743]. **(E–H)** Increased intrinsic neuronal activity in vDG neurons in the hippocampus of *Lrrc4c^–/–^* mice (2–4 months), as shown by increased current-firing curve, although resting membrane potential and input resistance are normal. *n* = 17 (4 mice) (WT), *n* = 13 (3) (KO), ^∗^*p* < 0.05, ^∗∗^*p* < 0.01, ns, not significant, Student’s *t*-test, two-way repeated measures ANOVA with Holm-Sidak multiple comparison test [**(E)** *t*_(__28__)_ = 1.3, *p* = 0.2041; **(F)** *t*_(__28__)_ = 0.7744, *p* = 0.4452; **(G)** interaction, *F*_(__17__,__476__)_ = 1.644, *p* = 0.0501; genotype, *F*_(__1__,__28__)_ = 0.69, *p* = 0.4132; current, *F*_(__17__,__476__)_ = 1174, *p* < 0.0001; **(H)** interaction, *F*_(__14__,__280__)_ = 5.189, *p* < 0.0001; genotype, *F*_(__1__,__20__)_ = 5.058, *p* = 0.0359; current, *F*_(__14__,__280__)_ = 504.9, *p* < 0.0001; 300 pA, *p* = 0.0198, 330 pA, *p* = 0.0380, 360 pA, *p* = 0.0049, 390 pA, *p* = 0.0069, 420 pA, *p* = 0.0016].

Lastly, we measured neuronal activity in the vDG, a brain region well-known to be associated with anxiety (Anacker and Hen, 2017). We found no alterations in sEPSCs or sIPSCs in *Lrrc4c^–/–^* vDG neurons (Figures 9A,B). In addition, there were no changes in miniature excitatory or inhibitory post-synaptic currents (mEPSCs or mIPSCs) in *Lrrc4c^–/–^* vDG neurons (Figures 9C,D), indicating that the normality of sEPSCs and sIPSCs in these neurons does not likely represent the consequences of network activity-dependent compensatory changes. Intriguingly, the activity of *Lrrc4c^–/–^* vDG neurons was increased, as indicated by the current-firing curve, although resting membrane potential and input resistance were normal (Figures 9E–H). These results suggest the possibility that neuronal activity is increased in the *Lrrc4c^–/–^* vDG under baseline and EPM conditions; however, this contrasts with the normal c-fos activity under baseline conditions and decreased c-fos activity under EPM conditions mentioned above (Figure 7K). Lastly, dDG neurons displayed normal neuronal excitability, as shown by resting membrane potential, input resistance, and current-firing curve (Supplementary Figure S2).

We also looked into changes in protein levels in total brain lysates and crude synaptosomes (P2) (Supplementary Figure S3). However, there were no genotype differences other than the ablation of NGL-1 in *Lrrc4c^–/–^* mice.

These results collectively suggest that NGL-1 deletion in ACC, MO, and vDG regions leads to distinct changes in excitatory synaptic transmission and neuronal activity, changes that are in line with the decreased c-fos signals in the *Lrrc4c^–/–^* MO, but not with those in the *Lrrc4c^–/–^* ACC and vDG (see section “Discussion” for potential reasons for these discrepancies).

## Discussion

Our present study shows that NGL-1 deletion in mice induces robust hyperactivity and anxiolytic-like behaviors. An analysis of c-fos expression revealed suppressed neuronal activity in various brain regions under baseline and EPM conditions. In addition, NGL-1-mutant neurons in cortical and hippocampal regions displayed distinct changes in synaptic transmission and intrinsic excitability. These changes in neuronal activity and synaptic and neuronal properties may contribute to the observed behavioral phenotypes in *Lrrc4c^–/–^* mice (summarized in Supplementary Figure S4).

*Lrrc4c^–/–^* mice displayed strong hyperactivity and anxiolytic-like behavior (Figures 1, 2). They also exhibited impairments in working memory (T-maze) and spatial learning and memory (Morris water maze), although novel-object-recognition memory and rotarod learning were normal (Figure 4). In contrast, these mice showed normal social interaction and social-novelty recognition and modestly decreased self-grooming (Figure 3). These results suggest that NGL-1 and related neural circuits are important for the maintenance of normal locomotor activity, anxiety-like behavior, and learning and memory. Notably, another mouse line that lacks netrin-G1, a cognate receptor for NGL-1 (Lin et al., 2003), shows overlapping behavioral phenotypes (Zhang et al., 2016b). Specifically, these latter mice show anxiolytic-like behavior in the EPM test and spatial learning and memory deficits in the Morris water-maze test, but normal levels of novel-object recognition and rotarod motor learning, similar to our mice. It is thus tempting to speculate that neuronal circuits involving the netrin-G1–NGL-1 trans-synaptic complex may contribute to the abnormal behaviors shared by *Lrrc4c^–/–^* and netrin-G1–mutant mice.

Whereas *Lrrc4c^–/–^* mice show impaired working memory in the T-maze and impaired spatial learning and memory in both initial and reversal phases of the Morris water maze, these mice showed normal novel-object recognition (Figure 4). The hippocampus has been associated with novel-object place or recency recognition, but not with novel-object-preference behavior (Barker and Warburton, 2011). In addition, working memory in the T-maze has been associated with brain regions including the hippocampus and prefrontal cortex (Spellman et al., 2015). Therefore, NGL-1 function in the hippocampus may contribute to the spatial and working deficits in *Lrrc4c^–/–^* mice. Indeed, a recent study on *Lrrc4c^–/–^* mice reported functional deficits in the hippocampus, including suppressed short-term synaptic plasticity in both the dorsal and ventral hippocampus (Choi et al., 2019).

Our c-fos results identified various brain regions in *Lrrc4c^–/–^* mice that display distinctly altered neuronal activities under baseline and EPM conditions (Figures 6, 7). These brains regions were frequently associated with those with strong NGL-1 expression that we identified by X-gal analyses (Choi et al., 2019). Specifically, lower baseline neuronal activities were observed in *Lrrc4c^–/–^* cortical and subcortical regions (ACC, MO, ILA, PIR2, EPd, LS, RSP, PVT, and dDG/dCA3/dCA1). In addition, EPM stimulation failed to increase neuronal activity in many *Lrrc4c^–/–^* brain regions, including the ACC, MO, ILA, EPd, and LS. The combination of these two properties—low baseline neuronal activity and dampened increase in neuronal activity upon EPM stimulation—in certain brain regions such as the ACC and MO seemed to markedly decrease final c-fos levels. These changes in neuronal activity under basal and EPM conditions may contribute to the anxiolytic-like behavior of *Lrrc4c^–/–^* mice. Intriguingly, c-fos levels in some other *Lrrc4c^–/–^* brain regions (dCA3, dCA1, RSP, and PVT) with low baseline c-fos levels were further decreased (rather than increased) by EPM stimulation, similar to results in WT mice, ultimately maintaining lower c-fos levels in these *Lrrc4c^–/–^* regions (Figures 7L–O). These changes may also contribute to the anxiolytic-like behaviors in *Lrrc4c^–/–^* mice. Notably, a recent study identified dCA3 as a brain region in netrin-G1-mutant mice that shows suppressed neuronal activation upon EPM stimulation (Zhang et al., 2016c), results similar to ours (Figure 7M).

The brain regions that we identified in the present study overlap with those previously reported to be associated with anxiety, including the ACC, LS, BNST, hippocampus, PVT, and BLA (Adhikari, 2014; Apps and Strata, 2015; Calhoon and Tye, 2015; Duval et al., 2015; Tovote et al., 2015). For instance, neuronal activity in both the ACC and MO has been positively correlated with the expression of anxiety (Wassermann et al., 2001; Kim et al., 2011). In addition, optogenetic activation of ACA neurons has been shown to induce anxio-depressive behavior (Barthas et al., 2015). The PVT projects densely to the nucleus accumbens as well as the dorsolateral region of the BNST (Li and Kirouac, 2008; Hsu and Price, 2009), known to be associated with anxiety (Duvarci et al., 2009; Kim et al., 2013; Avery et al., 2016).

Of the many brain regions that showed decreases in neuronal activity under baseline and EPM conditions, the ACC, MO, and vDG were further analyzed for synaptic transmission and the intrinsic excitability of neurons, based on their known relevance to anxiety and stronger changes in c-fos levels (Figures 8, 9). Layer 2/3 pyramidal neurons in the *Lrrc4c^–/–^* MO showed decreased intrinsic excitability, but unaltered excitatory and inhibitory synaptic transmission (Figures 8G–L), in line with the lower c-fos levels in the *Lrrc4c^–/–^* MO region under baseline and EPM conditions (Figures 6A–E, 7D–F). In contrast, layer 2/3 pyramidal neurons in the *Lrrc4c^–/–^* ACC showed normal intrinsic excitability, but increased (not decreased) frequency of sEPSCs (Figures 8A–F), a finding that appears to be at odds with the fact that NGL-1 is an excitatory post-synaptic adhesion molecule (Woo et al., 2009b) and with the lower c-fos levels in the *Lrrc4c^–/–^* ACC region under baseline and EPM conditions (Figures 6A–C, 7A–C). Moreover, vDG neurons in the *Lrrc4c^–/–^* hippocampus showed normal synaptic transmission, but increased (not decreased) intrinsic excitability (Figure 9), again, a finding that is not in agreement with the normal c-fos levels in the *Lrrc4c^–/–^* vDG region under baseline conditions or the lower c-fos levels in this region under EPM conditions (Figures 6F, 7K).

Several possible explanations can be put forward to account for these discrepancies. First, the synaptic and neuronal properties measured in slice preparations may not fully reflect the *in vivo* situation, suggesting the possibility that the slice preparation lacks a substantial portion of excitatory and inhibitory inputs and thus may not represent c-fos conditions *in vivo* at baseline. Second, the observed c-fos activity may be derived not only from excitatory neurons but also inhibitory neurons, whereas our electrophysiological recordings were made only in excitatory neurons. Third, it could be that we used layer 2/3 pyramidal neurons for our recordings, whereas c-fos activity in a cortical area may come from all cortical layers. Lastly, the changes in c-fos levels in a group of neurons may reflect the changes in upstream neurons that are caused by the loss of NGL-1 at excitatory synapses.

NGL-1 has been implicated in bipolar disorder (Greenwood et al., 2012) as well as ASD and developmental delay (Maussion et al., 2017). In addition, netrin-G1 and CDKL5, known to bind NGL-1, have been implicated in various brain disorders—netrin-G1 with Rett syndrome, ASD, schizophrenia and bipolar disorder (Borg et al., 2005; Archer et al., 2006; Nectoux et al., 2007; Eastwood and Harrison, 2008; Ohtsuki et al., 2008; O’Roak et al., 2012), and CDKL5 with Rett syndrome, epilepsy, intellectual disability, and ASD (Posar et al., 2015; Zhou et al., 2017; Zhu and Xiong, 2019). Many of these NGL-1-related disorders, including intellectual disability, schizophrenia, ASD, bipolar disorder, and Rett syndrome, involve cognitive dysfunctions and altered anxiety (Kim et al., 2000; Simon et al., 2004; Chahrour and Zoghbi, 2007). Therefore, the observed synaptic, neuronal, and behavioral phenotypes of *Lrrc4c^–/–^* mice may form a useful basis for future studies.

## Conclusion

Our results indicate that NGL-1 is important for the maintenance of normal locomotion, anxiety-like behavior, and learning and memory behaviors in mice. In addition, our results identify various brain regions that show suppressed neuronal activities under baseline and anxiety-inducing conditions involving altered synaptic and neuronal properties, which may underlie the altered behavioral phenotypes in NGL-1–mutant mice.

## Data Availability Statement

The datasets generated for this study are available on request to the corresponding author.

## Ethics Statement

The animal study was reviewed and approved by Committee of Animal Research at KAIST.

## Author Contributions

YC, HP, HK, SKa, and Y-EC performed behavioral experiment. YC, HP, SKa, HK, and HJ performed c-fos analysis. YC, HP, SKa, SKi, and SL performed electrophysiological experiment. YC and HJ performed Western blot analysis. IC performed the visual discrimination test. EK and S-HL designed the research and wrote the manuscript.

## Conflict of Interest

The authors declare that the research was conducted in the absence of any commercial or financial relationships that could be construed as a potential conflict of interest.

## Supplementary Material

FIGURE S1 Representative c-fos images in WT and *Lrrc4c^–/–^* mice. **(A–F)** Representative images of WT and *Lrrc4c^–/–^* mice under control and EPM conditions. The images show c-fos signals only (not NeuN). **(A)** (a) MOs + p (secondary and primary motor area), (b) ACC (anterior cingulate area or ACA cortex), (c) ILA (infralimbic area), (d) CP (caudoputamen), (e) LS (lateral septum), (f) MS (medial septal nucleus), (g) EPd (dorsal endopiriform nucleus), (h) PIR2 (pyramidal layer of piriform area). **(B)** (a) MOs + p, (b) ACC, (c) LS, (d) CP, (e) HY (hypothalamus), (f) MS, (g) EPd, (h) PIR2. **(C)** (a) MOs + p, (b) ACC, (c) LS, (d) CP, (e) BNSTpr (posterior bed nuclei of the stria terminalis), (f) BNSTal (anterolateral bed nuclei of the stria terminalis), (g) PIR2. **(D)** (a) RSP, (b) MOs + p, (c) SSP (Trunk), (d) CA3, (e) DG, (f) MD. **(E)** (a) RSP, (b) MOs + p, (c) SSp (Trunk), (d) CA1, (e) CA3, (f) DG, (g) MH, (h) LH, (i) PVT, (j) LA, (k) BLA. **(F)** (a) CA1, (b) CA3, (c) DG.

FIGURE S2 Normal synaptic transmission and intrinsic excitability in *Lrrc4c^–/–^* dDG neurons. **(A–D)** Normal intrinsic neuronal activity in dDG neurons in the hippocampus of *Lrrc4c^–/–^* mice (2–4 months), as shown by resting membrane potential, input resistance, and current-firing curve. *n* = 17 (4 mice) (WT), *n* = 23 (4) (KO), ns, not significant, Student’s *t*-test, two-way repeated measures ANOVA [**(A)** *t*_(38)_ = 0.8662, *p* = 0.3918; **(B)** *t*_(38)_ = 0.891, *p* = 0.3786; **(C)** interaction, *F*_(17,__646)_ = 1.983, *p* = 0.0105; genotype, *F*_(1,__38)_ = 0.1067, *p* = 0.7457; current, *F*_(17,__646)_ = 828.4, *p* < 0.0001; **(D)** interaction, *F*_(7,__245)_ = 1.002, *p* = 0.4303; genotype, *F*_(1,35)_ = 1.487, *p* = 0.2309; current, *F*_(7,__245)_ = 270, *p* < 0.0001].

FIGURE S3 Normal protein levels in total lysates and crude synaptosomes in the *Lrrc4c^–/–^* brain. **(A,B)** Normal protein levels in total lysates **(A)** and crude synaptosomes (P2) from whole brains of *Lrrc4c^–/–^* mice. *n* = 4 (WT/W), *n* = 4 (KO/K), ns, not significant, Student’s *t*-test.

FIGURE S4 Schema of the main findings. *Lrrc4c^–/–^* mice display hyperactivity, decreased anxiety-like behaviors, and impaired learning and memory as major behavioral phenotypes. We focused on the decreased anxiety, and found that anxiety-inducing stimuli (elevated plus-maze) lead to a significant increase in neuronal activity (as measured by c-fos levels) in WT mice, but this exposure-dependent increase is substantially blunted in *Lrrc4c^–/–^* mice.
