# Supplementary figures and images for: NGL-1/LRRC4C-Mutant Mice Display Hyperactivity and Anxiolytic-Like Behavior Associated With Widespread Suppression of Neuronal Activity
Source: Front Mol Neurosci. 2019 Oct 11;12:250. doi: 10.3389/fnmol.2019.00250 (PMC6798069; doi:10.3389/fnmol.2019.00250)

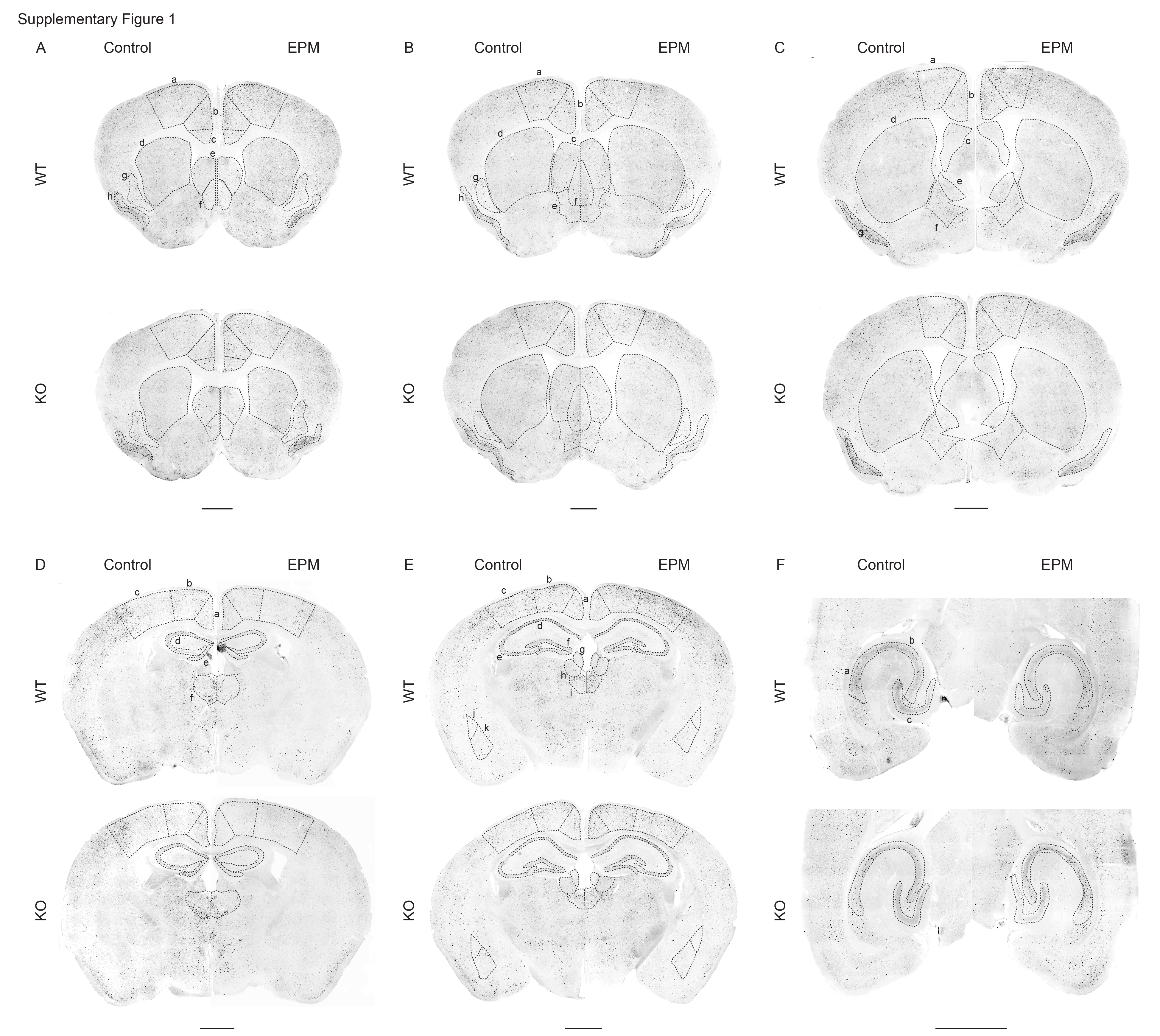

Supplement: FIGURE S1 — Representative c-fos images in WT and Lrrc4c–/– mice. (A–F) Representative images of WT and Lrrc4c–/– mice under control and EPM conditions. The images show c-fos signals only (not NeuN). (A) (a) MOs + p (secondary and primary motor area), (b) ACC (anterior cingulate area or ACA cortex), (c) ILA (infralimbic area), (d) CP (caudoputamen), (e) LS (lateral septum), (f) MS (medial septal nucleus), (g) EPd (dorsal endopiriform nucleus), (h) PIR2 (pyramidal layer of piriform area). (B) (a) MOs + p, (b) ACC, (c) LS, (d) CP, (e) HY (hypothalamus), (f) MS, (g) EPd, (h) PIR2. (C) (a) MOs + p, (b) ACC, (c) LS, (d) CP, (e) BNSTpr (posterior bed nuclei of the stria terminalis), (f) BNSTal (anterolateral bed nuclei of the stria terminalis), (g) PIR2. (D) (a) RSP, (b) MOs + p, (c) SSP (Trunk), (d) CA3, (e) DG, (f) MD. (E) (a) RSP, (b) MOs + p, (c) SSp (Trunk), (d) CA1, (e) CA3, (f) DG, (g) MH, (h) LH, (i) PVT, (j) LA, (k) BLA. (F) (a) CA1, (b) CA3, (c) DG. [file Image_1.JPEG]

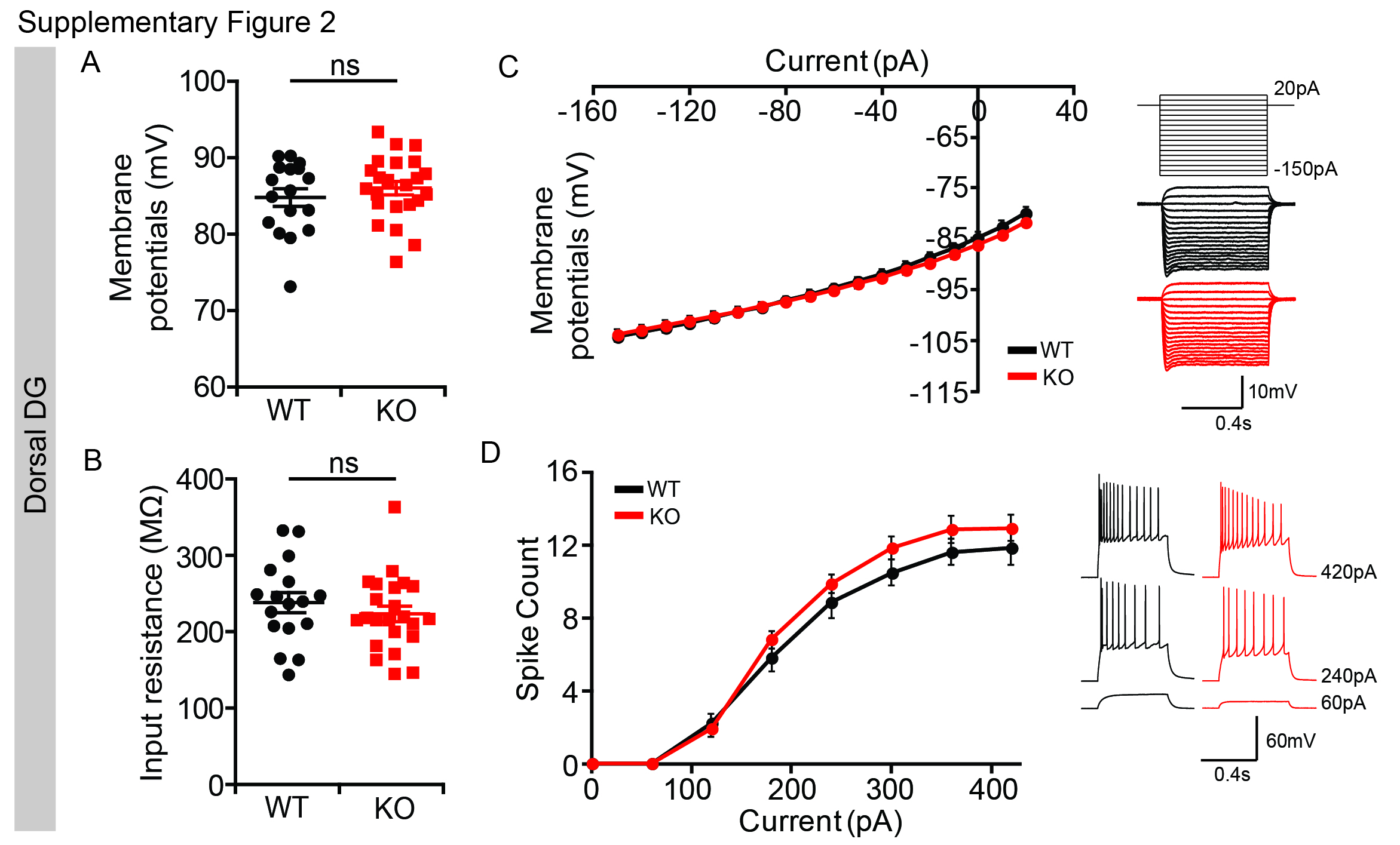

Supplement: FIGURE S2 — Normal synaptic transmission and intrinsic excitability in Lrrc4c–/– dDG neurons. (A–D) Normal intrinsic neuronal activity in dDG neurons in the hippocampus of Lrrc4c–/– mice (2–4 months), as shown by resting membrane potential, input resistance, and current-firing curve. n = 17 (4 mice) (WT), n = 23 (4) (KO), ns, not significant, Student’s t-test, two-way repeated measures ANOVA [(A) t(38) = 0.8662, p = 0.3918; (B) t(38) = 0.891, p = 0.3786; (C) interaction, F(17,646) = 1.983, p = 0.0105; genotype, F(1,38) = 0.1067, p = 0.7457; current, F(17,646) = 828.4, p < 0.0001; (D) interaction, F(7,245) = 1.002, p = 0.4303; genotype, F(1,35) = 1.487, p = 0.2309; current, F(7,245) = 270, p < 0.0001]. [file Image_2.JPEG]

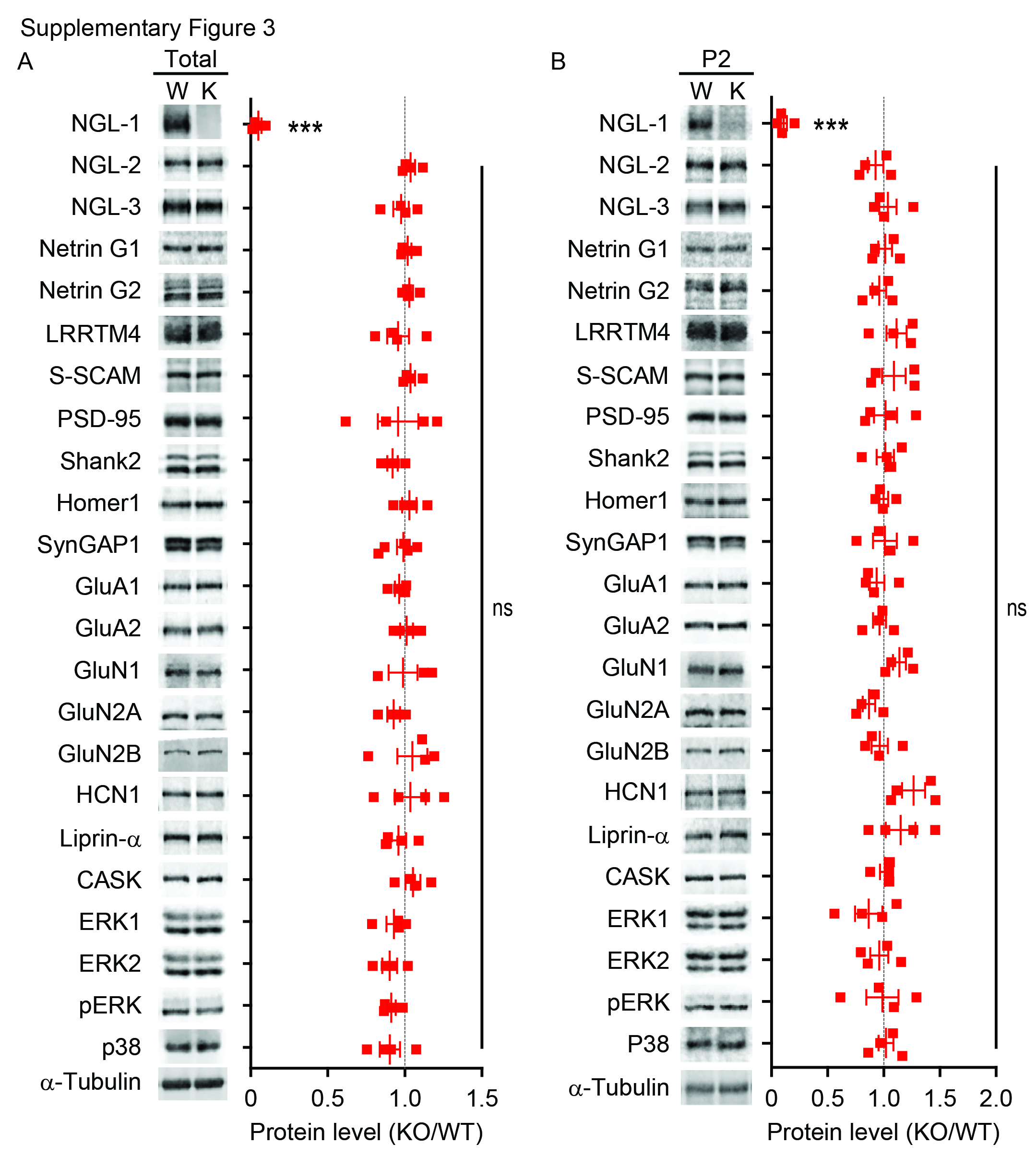

Supplement: FIGURE S3 — Normal protein levels in total lysates and crude synaptosomes in the Lrrc4c–/– brain. (A,B) Normal protein levels in total lysates (A) and crude synaptosomes (P2) from whole brains of Lrrc4c–/– mice. n = 4 (WT/W), n = 4 (KO/K), ns, not significant, Student’s t-test. [file Image_3.JPEG]

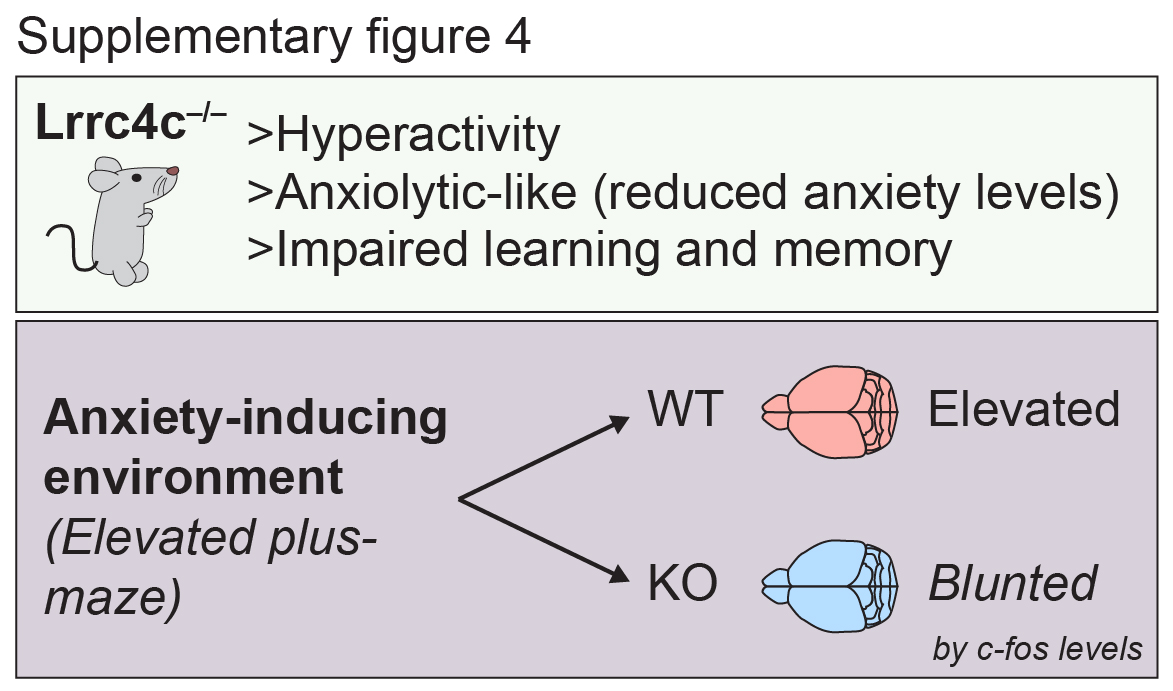

Supplement: FIGURE S4 — Schema of the main findings. Lrrc4c–/– mice display hyperactivity, decreased anxiety-like behaviors, and impaired learning and memory as major behavioral phenotypes. We focused on the decreased anxiety, and found that anxiety-inducing stimuli (elevated plus-maze) lead to a significant increase in neuronal activity (as measured by c-fos levels) in WT mice, but this exposure-dependent increase is substantially blunted in Lrrc4c–/– mice. [file Image_4.JPEG]
